# Immunotherapeutic targeting of aging‐associated isoDGR motif in chronic lung inflammation

**DOI:** 10.1111/acel.14425

**Published:** 2025-01-05

**Authors:** Pazhanichamy Kalailingam, SoFong Cam Ngan, Ranjith Iyappan, Afra Nehchiri, Khalilatul‐Hanisah Mohd‐Kahliab, Benjamin Sian Teck Lee, Bhargy Sharma, Radek Machan, Sint Thida Bo, Emma S. Chambers, Val A. Fajardo, Rebecca E. K. Macpherson, Jian Liu, Panagiota Klentrou, Evangelia Litsa Tsiani, Kah Leong Lim, I. Hsin Su, Yong‐Gui Gao, A. Mark Richar, Raj N. Kalaria, Christopher P. Chen, Cynthia Balion, Dominique de Kleijn, Neil E. McCarthy, Siu Kwan Sze

**Affiliations:** ^1^ Center for Genomic Medicine Massachusetts General Hospital and Harvard Medical School Boston Massachusetts USA; ^2^ School of Biological Sciences Nanyang Technological University Singapore Singapore; ^3^ Faculty of Applied Health Sciences Brock University St. Catharines Ontario Canada; ^4^ SCELSE Nanyang Technological University Singapore Singapore; ^5^ Centre for Immunobiology, the Blizard Institute, Bart's and the London School of Medicine and Dentistry Queen Mary University of London London UK; ^6^ Lee Kong Chian School of Medicine Nanyang Technological University Singapore Singapore; ^7^ Cardiovascular Research Institute National University Health System Singapore Singapore; ^8^ Institute of Neuroscience, Campus for Ageing and Vitality Newcastle University Newcastle upon Tyne UK; ^9^ Memory, Aging and Cognition Centre National University Health System Singapore Singapore; ^10^ Department of Pathology and Molecular Medicine McMaster University Hamilton Ontario Canada; ^11^ Department of Vascular Surgery UMC Utrecht Utrecht The Netherlands

**Keywords:** antibody, immune clearance, immunotherapy, inflammation, isoDGR, lung fibrosis

## Abstract

Accumulation of damaged biomolecules in body tissues is the primary cause of aging and age‐related chronic diseases. Since this damage often occurs spontaneously, it has traditionally been regarded as untreatable, with typical therapeutic strategies targeting genes or enzymes being ineffective in this domain. In this report, we demonstrate that an antibody targeting the isoDGR damage motif in lung tissue can guide immune clearance of harmful damaged proteins in vivo, effectively reducing age‐linked lung inflammation. We observed age‐dependent accumulation of the isoDGR motif in human lung tissues, as well as an 8‐fold increase in isoDGR‐damaged proteins in lung fibrotic tissues compared with healthy tissue. This increase was accompanied by marked infiltration of CD68+/CD11b + macrophages, consistent with a role for isoDGR in promoting chronic inflammation. We therefore assessed isoDGR function in mice that were either naturally aged or lacked the isoDGR repair enzyme. IsoDGR‐protein accumulation in mouse lung tissue was strongly correlated with chronic inflammation, pulmonary edema, and hypoxemia. This accumulation also induced mitochondrial and ribosomal dysfunction, in addition to features of cellular senescence, thereby contributing to progressive lung damage over time. Importantly, treatment with anti‐isoDGR antibody was able to reduce these molecular features of disease and significantly reduced lung pathology in vivo.

## Graphical abstract

Accumulation of isoDGR‐damaged proteins in aged lung tissues leads to macrophage infiltration and chronic inflammation. Anti‐isoDGR antibodies treatment clears isoDGR proteins and reduces inflammation and lung damage.

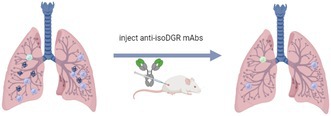

**Abbreviations**
AM: alveolar macrophage
BSA: bovine serum albumin
COPD: chronic obstructive pulmonary disease
DEG: differentially expressed gene
DPM: degenerative protein modification
DVC: digital ventilated cage
ECAR: extracellular acidification
ECM: extracellular matrix
ETC: electron transport chain
FN: Fibronectin
FPKM: fragments per kilobase of exon per million mapped reads
IM: interstitial macrophage
isoDGR: isoAsp‐Gly‐Arg
mAb: monoclonal antibody
MRI: magnetic resonance imaging
NGR: Asn‐Gly‐Arg
OCR: oxygen consumption rate
PBST: phosphate‐buffered saline with 0.5% Tween‐20
PBS‐TT: phosphate‐buffered saline with 0.5% Tween‐20, 0.1% Triton X‐100
PCA: principal component analysis
Pcmt1: protein‐L‐isoaspartate (D‐aspartate) O‐methyltransferase
RGD: Arg‐Gly‐Asp
ROS: reactive oxygen species
SPF: specific pathogen‐free
TMA: tissue microarray
VRW: voluntary running wheel
WB: western blot
WT: wild type

## 1 INTRODUCTION

At a fundamental level, aging results from the accumulation of molecular damage including degenerative protein modifications (DPMs) within body tissues, leading to gradual deterioration of tissue function and increased vulnerability to disease and death (Clarke, 2003; da Costa et al., 2016; Gladyshev et al., 2021; Kalailingam et al., 2023; López‐Otín et al., 2013; Robinson & Robinson, 2001; Schneider et al., 2021). Protein damage occurs spontaneously and has long been viewed as untreatable molecular culprits, since typical therapeutic strategies targeting genes or enzymes are not effective in this domain. We previously reported that age‐linked damage to the amino acid sequence NGR (Asn‐Gly‐Arg) of extracellular matrix (ECM) results in a ‘gain‐of‐function’ conformational switching to isoDGR motifs (isoAsp‐Gly‐Arg) that can mediate integrin binding (Cheow et al., 2013; Dutta et al., 2017; Park et al., 2021). We observed that extracellular isoDGR accumulates in body tissues from elderly human patients and in animal models of aging, while functional experiments confirmed that this motif binds to integrins on the surface of macrophages, initiating an outside‐in signaling cascade triggers expression and secretion of proinflammatory cytokines (Juang et al., 2017; Kim et al., 2018; Qin et al., 2015). To investigate further, we developed an isoDGR‐specific monoclonal antibody (isoDGR‐mAb), which was able to significantly extend both lifespan and healthspan in treated mice (Kalailingam et al., 2023; Park et al., 2021). However, the specific tissues and organ systems involved in this remarkable finding have not been identified, and the physiological relevance to elderly human patients has remained unclear.

Advancing age is the primary risk factor for significant lung pathology and associated mortality (Schneider et al., 2021). Numerous studies have highlighted that pulmonary function, crucial for supplying oxygen to body tissues, strongly predicts human morbidity and mortality (Cuttica et al., 2015; Duong et al., 2022; Lange et al., 2015; McAllister et al., 2007; Putman et al., 2016; Synn et al., 2021). Human lungs exhibit the largest surface area in the body, comprising hundreds of millions of alveolar sacs and an extensive capillary blood vessel network that facilitate rapid oxygen exchange (Schneider et al., 2021). The lung is supported by a scaffold formed of ECM components and resident interstitial cells that regulate pulmonary functions and local immune responses, including epithelial cells, alveolar macrophages (AMs), and peribronchial interstitial macrophages (IMs) (Schneider et al., 2021). Pulmonary ECM proteins exhibit slow turnover and are therefore susceptible to the accumulation of molecular damage that contributes to lung aging (Hackett & Osei, 2021) including structural and functional changes such as loss of elasticity, increased tissue inflammation, and reduced muscle strength. Accordingly, dysregulation of pulmonary ECM turnover has been shown to induce chronic inflammation of the airways and been implicated in the development of respiratory diseases (Boyd et al., 2020) including emphysema, chronic obstructive pulmonary disease (COPD), asthma, fibrosis, and acute respiratory distress syndrome (Burgstaller et al., 2017; Genschmer et al., 2019; Schneider et al., 2021).

IsoDGR modification has previously been observed in structural proteins such as fibronectin, laminin, tenascin C, and several other ECM constituents of human arteries, leading to increased leukocyte infiltration of coronary vessels (Dutta et al., 2017; Park et al., 2021). These ECM proteins are also essential components of human lungs, which consist of a complex anatomy of fibrous proteins (collagen, elastin), glycoproteins (fibronectin, laminin), glycosaminoglycans (heparin, hyaluronic acid), and proteoglycans (perlecan, versican) (Hackett & Osei, 2021). These long‐lived lung proteins are particularly susceptible to isoDGR accumulation, potentially triggering macrophage infiltration and expression of pro‐inflammatory cytokines (Park et al., 2021). Indeed, isoDGR structurally mimics the RGD integrin binding motif, and may therefore mediate leukocyte recruitment to induce pulmonary ‘inflamm‐aging’, but it is unknown whether this motif drives age‐linked lung diseases such as fibrosis and emphysema (Henderson et al., 2013, 2020; Morris et al., 2003; Munger et al., 1999; Slack et al., 2022).

In this study, we demonstrate that age‐associated deposition of isoDGR in lung ECM activates monocyte‐macrophages, thereby promoting chronic inflammation and eventual pulmonary dysfunction. We investigated isoDGR accumulation in human lung tissues microarray (TMA) obtained from individuals across various age groups and background, as well as from patients diagnosed with lung fibrosis. By analysing human lung tissue microarrays using immunofluorescence, we observed an age‐dependent accumulation of the isoDGR motif, which was 8‐fold enriched in fibrotic areas and accompanied by marked infiltration of CD68+/CD11b + macrophages. Next, we explored the mechanistic link between isoDGR accumulation and age‐associated pulmonary disease in both naturally aged mice and animals lacking the isoDGR repair enzyme Pcmt1. We observed significant accumulation of isoDGR‐modified proteins in murine lungs, accompanied by pulmonary inflammation, enlarged airspaces, tissue edema, congested blood vessels, and pronounced hypoxemia. Transcriptomic and functional analyses further revealed that isoDGR dysregulates ribosome activity, electron transport chain (ETC), and core mitochondrial functions, while also promoting the generation of reactive oxygen species (ROS) and driving senescence of lung cells. Conversely, treatment with an isoDGR‐specific antibody successfully reduced lung inflammation and pathology, in addition to alleviating redox stress and mitochondrial dysfunction in vivo. Together, these results demonstrate that an antibody directed against isoDGR is sufficient to trigger immune clearance of isoDGR‐damaged proteins from lung tissues, reduce inflammatory cytokine levels, and mitigate age‐linked pathology.

## 2 MATERIALS AND METHODS

### 2.1 Human lung tissue microarrays

Details of the isoDGR‐specific monoclonal antibody (isoDGR‐mAb) used in this study have been reported previously (Kalailingam et al., 2023; Park et al., 2021). To assess isoDGR accumulation in lung tissues, we obtained two types of paraffin‐embedded human lung tissue array (TMA‐LC2086a and TMA‐LC561) from Tissue Array LLC (Derwood, MD, USA). TMA‐LC2086a contains of 192 sections of normal human lung tissues with varying age, whereas TMA‐LC561 consists of 56 sections of pulmonary interstitial fibrosis tissues, alongside 2 cases each of cancer‐adjacent lung tissues and normal lung tissues. Our objective was to analyse isoDGR‐modified protein accumulation in aging human lung tissues and assess potential association with CD68+ and CD11b + cell distribution. To prepare the TMA slides for immunostaining, they were first deparaffinized and rehydrated by sequential immersion in 100% ethanol, followed by 75% and 50% ethanol, and finally PBS. Heat antigen retrieval was performed by placing the slides in a Pyrex beaker with antigen retrieval solution, and the temperature was raised to 95–99°C for 30–60 min. The beaker was covered with cling wrap to maintain temperature, and the slides were then removed and placed into a fresh coplin jar. After washing with milli‐Q H_2_O for 5 min, the slides were washed with PBS‐TT (0.5% Tween‐20, 0.1% Triton X‐100) for 15 min. Next, the slides were incubated with a protein block (2.5% normal goat serum, 1% BSA in 0.5% PBST) at room temperature for 20 min. After drying the slides, DAPI staining was applied for 15 min to determine tissue autofluorescence, which was later used to subtract tissue background from the antibody‐stained images. For immunostaining, the slides were treated with AlexaFluor‐conjugated primary antibodies (isoDGR‐AlexaFluor 488 [1:200] (homemade), CD68‐AlexaFluor 595 [1:200] (Cell signaling, 27,344), and CD11b‐AlexaFluor 700 (Invitrogen, 56–0118‐42)) overnight at 4°C. After immunostaining, the slides were washed three times using 1X PBS and mounted with aqueous mounting media. Finally, images were acquired using an Axion Scan.Z1 slide scanner for further analysis.

### 2.2 Animals

WT C57BL/6J mice at 17 months of age (catalog number: 000664) and Pcmt1^+/−^ (C57BL/6 background) mice (B6;129S4‐Pcmt1tm1Scl/J; Strain #:023343; RRID:IMSR_JAX:023343) were obtained from the Jackson Laboratory in Bar Harbor, ME, USA. Animals were housed under specific pathogen‐free (SPF) conditions in isolator cages for two weeks prior to the start of the experiments. The animal house maintained at room temperature of 24°C with a 12 h light/dark schedule. Mice were provided access to Purina rodent chow diet and tap water ad libitum. Mice deficient in the deamidation repair enzyme Pcmt1 were previously generated by Clarke and co‐worker (Kim et al., 1999). The Pcmt1^+/−^ (male and female) mice were bred to yield litters comprising Pcmt1^+/+^, Pcmt1^+/−^, and Pcmt1^−/−^ offspring in the expected Mendelian ratios. Mouse genotype was confirmed by PCR using the primers as shown in Table S1. All animal experiments used age‐ and sex‐matched mice unless otherwise specified. All mouse procedures were performed in a humane manner and approved by the Nanyang Technological University Institutional Animal Care and Use Committee (IACUC protocol # ARF‐SBS/NIE‐A18016, ARF‐SBS/NIE‐A19029) or the Animal Care Committee at Brock University (AUP # 22–08‐04).

### 2.3 Lung tissue H&E and immunostaining

Lungs from Pcmt1^+/+^, Pcmt1^+/−^, Pcmt1^−/−^, and mAb‐treated Pcmt1^−/−^ mice were collected and fixed with 4% PFA at 4°C for 24 h. Tissues were washed with 1X PBS and transferred into 15% sucrose, then 30% sucrose, and kept at 4°C. Lungs were subsequently embedded in OCT compound with dry ice and sectioned (10 μm) using a Leica CM3060S Cryostat. Sections were mounted onto Fisherbrand Superfrost Plus microscope slides, then kept in warm PBS for 20 min to remove OCT prior to staining with haematoxylin and eosin. Airspace enlargement in mouse lung sections was quantified using ImageJ's Analyze Particles function. Images were first converted to grayscale, and thresholding was applied to segment the airspace regions. The Analyze Particles function was then used with appropriate parameters (e.g., size and circularity) to filter particles and exclude noise. This process was repeated for multiple regions, the average airspace area was calculated, and the data recorded. For immunostaining, slides were permeabilized with 0.5% PBST for 2–3 h and then incubated with blocking buffer (2.5% normal goat serum, 1% BSA in 0.5% PBST) for 1 h at RT prior to addition of primary antibodies (isoDGR [1:200], fibronectin [1:200], Abcam ab2413 and CD68 [1:200], Abcam, ab283654) overnight at 4°C. Slides were washed with PBS (3X) for 5 min, then incubated with secondary antibodies conjugated to AlexaFluor 488 and 594 (1:500) for 1 h at RT. Immunostained slides were washed with PBS (3X) for 5 min then incubated with DAPI for 15 min to visualize cell nuclei. After staining, slides were washed with 1X PBS and mounted with aqueous mounting media. Images were acquired using a Zeiss LSM710 confocal microscope.

### 2.4 MRI analysis

Mouse lungs of different genotypes from the same litter were excised for MRI imaging. All MRI experiments were conducted on a 14 Tesla Bruker Ascend 600WB vertical magnet, equipped with a MicWB40 micro‐imaging probe and a Micro2.5 gradient system. A quadrature coil with an inner diameter of 30 mm (MICWB40 RES 600 1H 040/030) was used to transmit/receive MR signals. Images were obtained using Paravision v6.0.1 software. A modified multi‐spin‐multi‐echo (MSME) pulse sequence was used with an echo time (TE) of 8 ms, a repetition time (TR) of 3000 ms, with 2 averages to acquire images from the excised mouse lungs (positioned in a 30 mm diameter glass tube). The tissue was surrounded by Fomblin® to suppress background signals and placed horizontally such that axial images provided a cross‐section of both lobes. Each slice thickness was 0.4 mm, with a 22 × 22 mm field of view, and each scan was captured as a 512 × 512 pixelated image.

### 2.5 Western blot analysis

Western blot analysis was performed using standard methods. Primary antibodies and dilutions were as follows: isoDGR (mouse monoclonal 1:1000), Pcmt1 (rabbit polyclonal, Abcam 1:1000), and GAPDH (Invitrogen 1:1000).

### 2.6 SpO_2_ assay

We used a rodent oximeter to measure SpO2 in Pcmt1^+/+^, Pcmt*1* ^*+/−*^, Pcmt1^−/−^, and mAb‐treated Pcmt^−/−^ mice according to manufacturer's protocol.

### 2.7 Evans blue lung permeability assay

Mice were anaesthetized by isoflurane inhalation, and 200 μL 0.5% Evans blue in 1X PBS was administered via retro‐orbital route. After 30 min, mice were perfused with 15 mL of 1X PBS. Organs were collected and air dried to eliminate water content variability between different tissues. A 500 μL volume of formamide was added to 50–100 mg lung tissue and incubated at 55°C for 24 h to extract Evans blue. After removing tissue fragments from the sample tube, absorbance was measured at 610 nm using neat formamide as a blank. The final amount of Evans blue extravasation was calculated as ng dye per mg of tissue. The experiment was performed with *n* = 3 animals.

### 2.8 Collection of bronchoalveolar lavage (BAL) fluid for cytokine assay

Synthetic isoDGR‐peptide (100 μg) in 50 μL of PBS (or PBS only vehicle control) was intranasally injected into the lungs of Pcmt1^+/+^ mice (C57BL/6 background). After 24 h, mice were anaesthetized by ketamine/xylazine injection, and 200 μL PBS was used to lavage the lungs three times. Lavage fluid was then centrifuged, and the cell‐free supernatants collected for cytokine analysis.

### 2.9 Cytokine multiplex bead assay

The LEGENDplex^Tm^ mouse inflammation panel (Biolegend, San Diego, CA) was used to measure 13 different cytokines (IL‐23, IL‐1α, IL‐1β, IL‐6, IL‐10, IL‐12p70, IL‐17A, IL‐23, IL‐27, MCP1, IFN‐β, IFN‐γ, TNF‐α, and GM‐CSF) in plasma from Pcmt1^+/+^, Pcmt1^+/−^, Pcmt1^−/−^, and mAb‐treated Pcmt^−/−^ mice (assessed by LSRII flow cytometer according to the manufacturer's protocol).

### 2.10 Terminal deoxynucleotidyl transferase dUTP nick end labeling (TUNEL) assay

Lung sections were immersed in PBS for 5 min (2X) and incubated with proteinase K (1:100 in 10 mM Tris pH 8) for 20 min at room temperature. Slides were rinsed with PBS then incubated with equilibration buffer for 20 min. Sections were next incubated with labeling reaction mixture (equilibration buffer 40 μL, terminal deoxyribonucleotidyl transferase [TdT] enzyme buffer 5 μL, labeling solution 5 μL) at 37°C for 1 h. Slides were then washed with PBS (3X) for 5 min prior to incubation with DAPI for 10 min followed by a further PBS wash for 5 min. The slides were mounted with glycerol‐based mounting media. TUNEL‐positive apoptotic cells were observed, and images were acquired using a Zeiss LSM710 confocal microscope.

### 2.11 Senescence‐associated β‐galactosidase (SA‐βgal)

Cryosections of frozen lung tissue were fixed with 1% PFA for 10 min and washed in PBS (3× at 5 min/wash) then incubated overnight at 37°C with staining buffer (5‐bromo‐4‐chloro‐3‐indolyl β‐D‐galactopyranoside [X‐gal, Bioline, London, UK], 5 mm K3Fe[CN]_6_, 5 mm K4Fe[CN]_6_, and 2 mm MgCl_2_ in PBS at pH 6.0). After washing with PBS, the sections were mounted with glycerol‐based mounting media and imaged using bright field microscopy.

### 2.12 Cell culture and generation of Pcmt1 knock‐down cells by shRNA

HUVECs were cultured in DMEM containing 10% FBS and 1% penicillin–streptomycin at 37°C in a CO_2_ incubator. Pcmt1 shRNA in PLKO plasmid (GCGCTAGAACTTCTATTTGAT, TRCN0000036400, Sigma‐Merck USA) was transfected into HUVECs. After 24 h, puromycin (2 μg/mL) was used to select stably transformed cells. Pcmt1 knockdown was confirmed by western blot.

### 2.13 CellROX® ROS assay

A total of 12,000 HULEC‐5a cells were seeded into each chamber of a 96‐well plate (each chamber was coated with native FN, isoDGR‐FN, or PBS control in the presence or absence of 2 μg/mL anti‐isoDGR mAb). After 24 h, 5 μM CellROX red reagent (Thermo Fisher Scientific, Inc.) was added to the HULEC‐5a cultures and incubated for 30 min in a 5% CO_2_ incubator at 37°C. The cells were then washed with PBS, fixed with 3.7% formaldehyde in PBS for 15 min, and nuclei stained with DAPI for 5 min at room temperature. ROS positive cells were imaged using a Zeiss LSM710 confocal microscope.

### 2.14 Bleeding assay

Pcmt1^+/+^, Pcmt*1* ^−/−^, and Pcmt1^−/−^ + mAb mice were anesthetized with a mixture of ketamine and xylazine and placed in a prone position. A distal 5‐mm segment of the tail was amputated with a scalpel, and the tail was immersed in PBS at 37°C. Bleeding patterns were continuously monitored, and bleeding volume was measured based on changes in body weight. Sensitivity and extent of variation of bleeding time and volume were compared in mice treated with or without IsoDGR mAb.

### 2.15 RNA‐seq analysis

RNA‐seq was performed by Novoseq Technology, Singapore. Four libraries of transcriptomic samples were generated from the lungs of Pcmt1^*+/+*^, Pcmt1^*+/−*^, Pcmt1^*−/−*^, and mAb‐treated Pcmt1^*−/−*^ mice. The length of RNA fragments was detected using an Agilent 2100 Bioanalyzer (Agilent, USA). The cDNA library was then constructed using PCR amplification. RNA‐seq was carried out with the Novogene strategy on an Illumina second‐generation high‐throughput sequencing platform. Reads with inferior quality or adapters were filtered. Clean reads data were processed using Tophat2 and Cufflinks software to complete the alignment of transcriptomes and transcript splicing analysis separately. Clean reads were mapped to the *Mus musculus* reference genome (GRCm38.p6/NCBI, GCF_000001635.26).

### 2.16 Differentially expressed genes (DEG) analysis

Normalized fragments per kilobase of exon per million mapped reads (FPKM) was used to calculate gene expression level with the union counting model in HTSeq software (Princeton University, USA). We applied a cut‐off value of FPKM >1 to define gene expression, and DEG analysis was performed using DESeq (Bioconductor, USA). Genes with differential expression were screened, and hierarchical clustering analysis was performed.

### 2.17 Lung cell preparation and single cell RNA sequencing

Lung tissues were collected from 6‐week‐old Pcmt1^+/+^, Pcmt1^−/−^, and mAb‐treated Pcmt1^−/−^ mice then transferred into RPMI‐1640 medium containing 10% FBS and kept on ice. The tissues were washed with 1X PBS and then chopped into small pieces with scissors. Next, the tissue was resuspended in 1 mL digestion buffer consisting of collagenase type I (2 mg/mL), hyaluronidase (1 mg/mL), and DNase I (0.2 mg/mL) in RPMI‐1640 medium before incubation at 37°C for 30–40 min with gentle agitation at 110 RPM. The tissue suspension was then gently pipetted up and down, and the resulting cell suspension was passed through 100 μm mesh filters. The cell suspension was quenched with 10 mL of RPMI containing 10% FCS. After centrifugation at 500 ***g*** for 3 min at 4°C, the cell pellet was re‐suspended in RPMI medium containing 10% FBS for scRNA‐seq.

### 2.18 Single cell RNA sequencing and data analysis

Cells were subjected to droplet‐based single‐cell sequencing. The cDNA libraries were prepared using a Chromium Single Cell 3′ V3 kit (10X Genomics, USA) according to the manufacturer's instructions and then sequenced on a NovaSeq6000 (Illumina, USA). Raw sequencing reads were aligned to the mm10 (GENCODE vM23/Ensembl 98) mouse reference genome using Cell Ranger (v7.1.0) to generate single‐cell count matrices that were normalized, integrated, and annotated using Seurat (v4.30) (Hao et al., 2021). Low‐quality cells were filtered using uniform quality control thresholds; cells with RNA counts less than 300 and with mitochondrial read percentages more than 20% were filtered out. A total of 29,653 cells were obtained across all three conditions. Log normalization was implemented in Seurat. Principal component analysis (PCA) was performed to reduce the dimensionality of each dataset, and the first 35 PCA components were used to construct UMAP. Unsupervised cell clustering was performed with the Louvain algorithm. Cluster marker detection was performed by differentially expressed gene (DEG) analysis for each marker against the remaining markers using FindAllMarkers. Azimuth scRNA‐seq references for lung cells were downloaded from the LungMAP portal. Cell type annotation was performed automatically using LungMAP Cell Reference with Azimuth, (Guo, Morley, et al., 2022) since LungMap includes comprehensive annotation of endothelial cells, epithelial cells, mesenchymal cells, and immune cells in lung tissue (Sun et al., 2022).

### 2.19 Reverse transcription‐quantitative polymerase chain reaction (RT‐qPCR)

Total RNA isolated from lung tissues was treated with DNase and reverse‐transcribed using a first‐strand DNA synthesis kit from Invitrogen. PCR was performed on an ABI Fast 7500 System (Applied Biosystems, Foster City, CA). TaqMan probes for the respective genes were custom‐generated by Applied Biosystems based on sequences from the Illumina array and used as per the manufacturer's instructions. Expression levels of target genes were determined in triplicate from the standard curve and normalized to GAPDH mRNA level. The RT‐PCR primers are exhibited in Tables S2 and S3.

### 2.20 Seahorse assay for metabolic flux analysis and glycolysis stress test

The metabolic flux analyses and glycolysis stress test were performed using a Seahorse XF96 (Agilent Technologies, USA). In the metabolic flux analysis, oxygen consumption rate (OCR) was measured to assess mitochondrial function in HULEC‐5a cells. In brief, 12,000 cells per well were seeded into MCDB media (Merck, Singapore) using an XF96 well plate (Agilent, Santa Clara, CA) coated with PBS control, native FN, or isoDGR‐FN, in the presence or absence of 2 μg/mL anti‐isoDGR mAb. After 1 h, the medium was switched to Seahorse XF DMEM (Agilent), containing 10 ng/mL epidermal growth factor (EGF), 1 μg/mL hydrocortisone, and 10 mM glutamine, then incubated in a non‐CO_2_ incubator. The Seahorse XF96 Analyzer (Agilent) was used to measure extracellular acidification (ECAR) and oxygen consumption rate (OCR) in real time. The OCR assay consisted of three distinct injections. The first injection of 1 μM oligomycin blocks mitochondrial ATP production; the second injection of 1.5 μM FCCP (carbonyl cyanide‐4 (trifluoromethoxy) phenylhydrazone) uncouples the mitochondrial membrane to assess maximal cellular respiration; and the third injection of 100 μM rotenone together with 1 μM antimycin A blocks ETC complexes I and III, respectively. For glycolysis stress test, a similar procedure was performed except that non‐glycolytic acidification was measured first in the absence of both glucose and pyruvate. Final concentrations of 10 mM glucose (Sigma Aldrich, St. Louis, MO), 1 μM oligomycin (Sigma Aldrich), and 5 mM 2‐deoxy‐D‐glucose were added to assess ECAR.

### 2.21 Digital ventilated cage (DVC) and metabolic cage system monitoring

Naturally aged C57BL/6 mice (18–26 months old) were used in this study. Mice were randomly assigned to either the isoDGR‐mAb treatment or the control groups. The activity of the mice was continuously monitored in their home cages using the Digital Ventilated Cage (DVC) system. This system is equipped with an array of sensors that capture real‐time data on mouse activity, including locomotion, rearing, and overall cage exploration. Mice were housed individually in DVCs until they reached 26 months of age. To further assess the impact of isoDGR‐mAb on learning and physical activity, voluntary running wheels (VRW) were integrated into the DVC system. The VRW allowed mice to engage in voluntary exercise, with their running distance, speed, and duration continuously recorded. In addition to activity monitoring, mice were placed in Promethion metabolic cage systems to evaluate respiratory functions. Parameters such as oxygen intake (VO2) were measured continuously over a 24‐h period. Data were compared between the isoDGR‐mAb‐treated and control groups to assess the effects of treatment on respiratory function.

### 2.22 Statistical analysis

Statistical analyses were performed using GraphPad Prism v9.0 (GraphPad Software, Inc., San Diego, CA). Data were tested for normality using D'Agostino & Pearson or Shapiro–Wilk tests. For normally distributed data, differences between groups were assessed either by two‐tailed unpaired Student's *t* test or one‐way ANOVA for multiple groups, followed by Tukey's multiple comparisons test. Otherwise, the Kruskal–Wallis test with Dunn's multiple comparisons post‐hoc test was used (*p* < 0.05 was considered significant).

## 3 RESULTS

### 3.1 Advancing age and lung fibrosis are associated with isoDGR and macrophage accumulation

**FIGURE 1:**
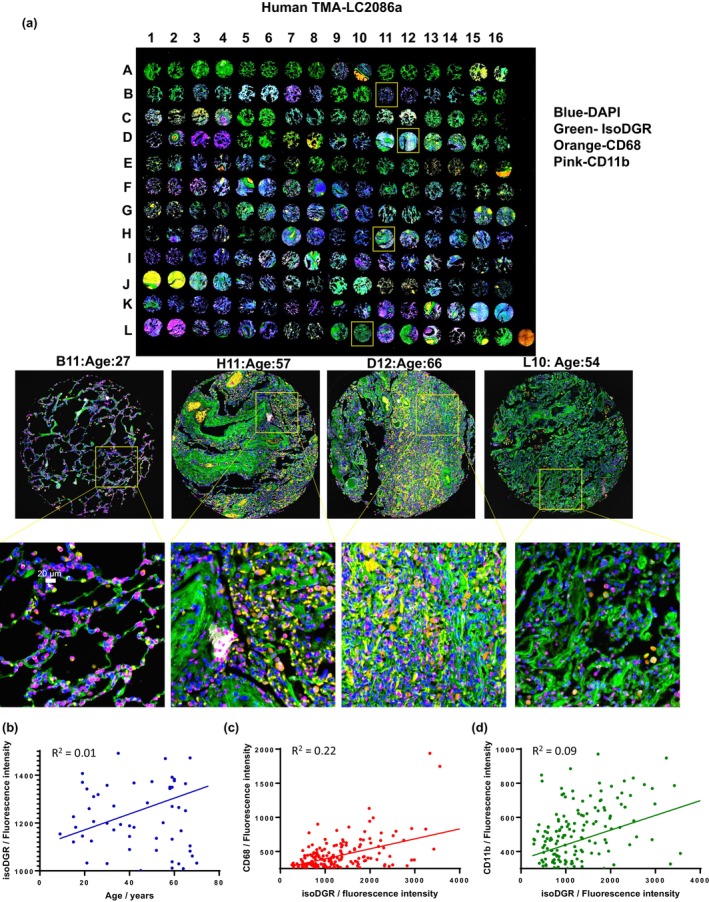
Analysis of immunohistochemistry of age‐induced accumulation of isoDGR‐modified proteins correlates with CD68+ and CD11b + cells in human lung tissue. (a) Representative immunostaining images showing isoDGR‐protein distribution and correlation with CD68+ and CD11b + immune cells in 192 sections of normal human lung tissues of varying age. Representative zoomed images are shown: B11: Age 27, H11: Age 57, D12: Age 66, L10: Age 54. (b) IsoDGR level was positively correlated with age (linear regression slope 3.32). (c) CD68 level was positively correlated with isoDGR level. (d) CD11b level was positively correlated with isoDGR level.

We initially assessed isoDGR‐modified protein levels in human lung using tissue microarrays to assess motif levels over life course. We examined two lung tissue microarrays (TMA‐LC2086a and TMA‐LC561), which included sections of healthy lung tissues from 192 individuals across various age groups and 56 sections of pulmonary interstitial fibrosis tissue. Immunostaining of isoDGR revealed high accumulation in aged lung and fibrotic areas in particular, accompanied by elevated frequencies of CD68+/CD11b + immune cells. Further analysis confirmed that isoDGR‐protein levels in normal human lung tissues were positively correlated with donor age irrespective of other known influences including genetics, lifestyle, and potential sociodemographic factors (Figure 1, isoDGR staining was positively correlated with age by linear regression; slope 3.32). We also detected CD68+ and CD11b + cells in most of the aging human lung tissue samples, and frequencies were positively correlated with isoDGR levels. These findings suggest that isoDGR‐modified protein damage increases with aging and is associated with immune cell infiltration, which may contribute to a range of age‐linked lung disorders. Indeed, in a separate analysis of tissues from pulmonary fibrosis cases and lung cancer patients, we observed that age‐related accumulation of isoDGR proteins was increased 8‐fold in disease settings (Figure S1, slope 26.20 by linear regression). Again, isoDGR levels were positively correlated with localization of CD68+ and CD11b + immune cells, indicating that isoDGR‐motif may contribute to macrophage activation and pro‐fibrotic lung inflammation. Statistical details of the linear regression results are shown in Tables S4 and S5. Normal lung tissues exhibited marked variability in isoDGR deposition, likely due to natural diversity in the general population, whereas the statistical results for fibrotic lung tissues showed a far stronger correlation. These data may suggest a role for isoDGR in the pathology of pulmonary fibrosis.

### 3.2 IsoDGR protein damage promotes age‐linked lung pathology

**FIGURE 2:**
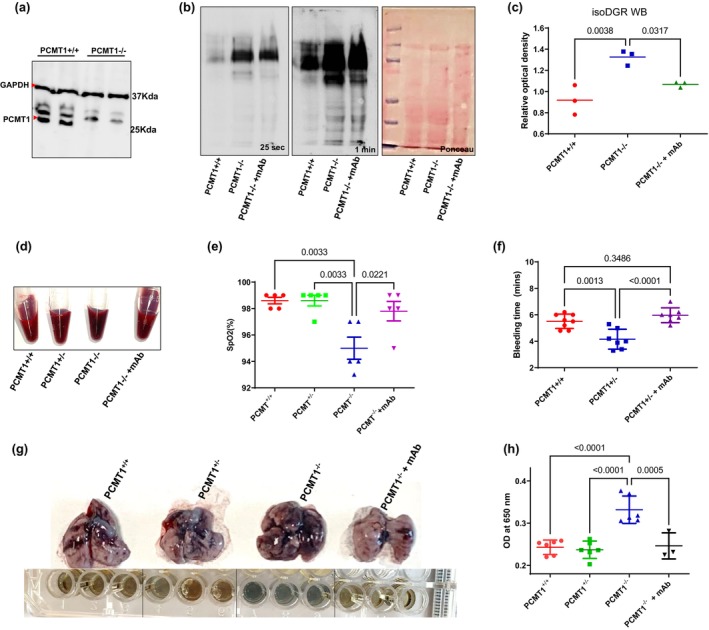
IsoDGR‐damaged protein accumulation in lungs impacts oxygen exchange. (a) Lung protein lysates from Pcmt1^+/+^ and Pcmt1^−/−^ mice were subjected to western blot using antibodies against Pcmt1 enzyme. GAPDH was used as a loading control (*n* = 3). (b) Lung protein lysates from Pcmt1^+/+^, Pcmt1^−/−^, and mAb‐treated Pcmt1^−/−^ mice were subjected to western blot using isoDGR‐specific mAb. Protein loading was visualized by Ponceau S. (*n* = 3) (c) Graph showing quantification of isoDGR‐damaged protein levels in the lungs of Pcmt1^+/+^, Pcmt1^−/−^, and mAb‐treated Pcmt1^−/−^ mice. (d) Representative images of blood samples from each phenotype collected at 6 weeks. Blood from Pcmt1^−/−^ mice appeared dark red in colour, unlike that of mAb‐treated Pcmt1^−/−^ mice. (e) Dot plot shows SpO2 level in WT, Pcmt1^+/−^, Pcmt1^−/−^, and mAb‐treated Pcmt1^−/−^ mice at 6 weeks (*n* = 5). SpO2 level was reduced in Pcmt1^−/−^ mice and markedly improved by mAb treatment. (f) Dot plot of tail bleeding time in Pcmt1^+/+^, Pcmt1^+/−^, and mAb‐treated Pcmt1^+/−^ mice (*n* = 7–8) at 1.5 years. (g) Dot plot of tail bleeding time in Pcmt1^+/+^, Pcmt1^−/−^, and mAb‐treated Pcmt1^−/−^ mice at 6 weeks (*n* = 6). (h) Representative lung appearance upon assessment of pulmonary vascular permeability by Evans blue dye extravasation assay. (i) Dot graph showing quantitative analysis of Evans blue‐labelled albumin extravasation from the lungs of Pcmt1^+/+^ (*n* = 6), Pcmt1^+/−^ (*n* = 6), Pcmt1^−/−^ (*n* = 6), and mAb‐treated Pcmt1^−/−^ mice (*n* = 3) assessed at 6 weeks. Statistical significance was assessed using one‐way ANOVA (c, f, g, i) or the Kruskal–Wallis test (e). Results are shown as mean ± SEM.

We next sought to confirm that isoDGR‐modified proteins can promote age‐linked lung pathology and assess whether this motif represents a promising target for novel immunotherapies. For this, we investigated the functional role of isoDGR in both naturally aged mice and animals that lack the corresponding repair enzyme Pcmt1. Deletion of Pcmt1 results in extensive accumulation of isoDGR protein damage in mouse body tissues (Lowenson et al., 2001) leading to premature aging and a reduced lifespan of just 4–12 weeks. (Kim et al., 1997, 1999; Yamamoto et al., 1998) In previous work, we showed that isoDGR interacts with macrophage integrins to induce pathological release of TNFα and MCP1, (Park et al., 2021) whereas weekly treatment with 1 mg/kg isoDGR‐specific mAb reduced vascular inflammation and extended the lifespan of prematurely aged Pcmt1^−/−^ mice. (Kalailingam et al., 2023) To investigate the pathological effects of isoDGR deposit across different organs, in this study we first generated global Pcmt1^−/−^ mice by crossing Pcmt1^+/−^ males with Pcmt1^+/−^ females (genotype was confirmed by PCR of tail genomic DNA; 25% of pups born were Pcmt1^−/−^ consistent with the expected Mendelian ratio). Pcmt1 deletion in mouse lung tissue was confirmed by western blot of protein extract from knockout and wild‐type animals (Figure 2a). Further western blot analysis indicated that isoDGR‐modified lung proteins accumulated to high levels in Pcmt1^−/−^ mice compared with WT control mice, and isoDGR‐specific mAb treatment reduced damaged protein levels in body tissues (Figure 2b,c). Examination on autopsy revealed severe pulmonary edema in mice that died at 4–6 weeks (10%), moderate pathology in mice that died at 6–10 weeks (60%), and less severe histology in mice that survived beyond 10 weeks (30%). Moreover, accumulation of isoDGR‐damaged proteins was also observed in naturally aged WT mice (17 months old) and motif levels were reduced by target‐specific mAb immunotherapy but not isotype‐matched antibody treatment (Figure S2).

### 3.3 IsoDGR‐induced tissue damage impairs pulmonary oxygen exchange

Having observed pulmonary edema in Pcmt1^−/−^ mice, we next performed hematology examinations to better determine the mechanisms underlying isoDGR‐induced lung pathology. Blood from Pcmt1^−/−^ and older Pcmt1^+/−^ animals was immediately noted as being darker in shade than that of Pcmt1^+/+^ mice, indicating marked accumulation of carboxyhemoglobin due to hypoxemia (Figure 2d). We therefore proceeded to check blood oxygen (SpO_2_) levels in Pcmt1^+/−^, Pcmt1^−/−^, mAb‐treated Pcmt1^−/−^ mice, and Pcmt1^+/+^ controls. These data indicated a significant reduction of SpO_2_ in Pcmt1^−/−^ mice (94%–97%) compared with Pcmt1^+/+^ control mice (97%–99%), suggesting significant hypoxemia in animals that lack the isoDGR repair enzyme (Figure 2e). Given that this pathology can arise from either impaired lung function or haematological defects, we next performed a complete blood count to evaluate the likely cause of hypoxemia. RBC counts and hemoglobin levels were not significantly different between Pcmt1^−/−^ and Pcmt1^+/+^ mice, suggesting that these parameters did not play an important role in the observed hypoxemia (Figure S3). Having previously identified that isoDGR‐modified fibrinogen may promote coagulation in blood from cardiovascular disease (CVD) patients, we next performed tail bleeding assays in Pcmt1^+/−^ and Pcmt1^+/+^ mice at 1.5 years of age (by which time isoDGR‐modified protein levels are significantly increased in Pcmt1^+/−^ compared to Pcmt1^+/+^ animals). Intriguingly, isoDGR accumulation was found to be associated with pro‐thrombotic phenotypes, as demonstrated by a significantly reduced bleeding time in Pcmt1^+/−^ mice compared with age‐matched Pcmt1^+/+^ mice (Figure 2f). Next, we performed a bleeding assay in Pcmt1^+/+^, Pcmt1^−/−^, and mAb‐treated Pcmt1^−/−^ mice. We observed a significantly shorter bleeding time in Pcmt1^−/−^ mice compared to Pcmt1^+/+^ animals. Treatment with isoDGR motif‐specific mAb restored bleeding time to levels comparable with Pcmt1^+/+^ mice. These results suggest that excess isoDGR‐protein in body tissues is associated with pro‐thrombotic phenotypes in Pcmt1^−/−^ mice. We therefore proceeded to test extravasation of Evans blue dye from the pulmonary blood vessels of Pcmt1^+/+^, Pcmt1^+/−^, Pcmt1^−/−^, and mAb‐treated Pcmt1^−/−^ mice. We observed a marked increase in Evans blue extravasation from the lungs of Pcmt1^−/−^ mice, indicating significant vascular leakage consistent with pulmonary oedema, impaired oxygen exchange, and resultant hypoxemia. Crucially, isoDGR‐specific mAb treatment restored blood samples to a bright red color typical of Pcmt1^+/+^ oxygenation levels (Figure 2d) and normalised SpO_2_ values (Figure 2e) while also reducing coagulopathy (Figure 2f,g) and minimising vascular leakage (Figure 2h,i). Together, these results indicate that isoDGR‐induced lung pathology and coagulopathy are the primary causes of hypoxemia in Pcmt1^−/−^ mice and that isoDGR‐specific immunotherapy can ameliorate these pathological features in vivo.

### 3.4 IsoDGR‐enriched lung tissues display histological features of age‐linked pathology

**FIGURE 3:**
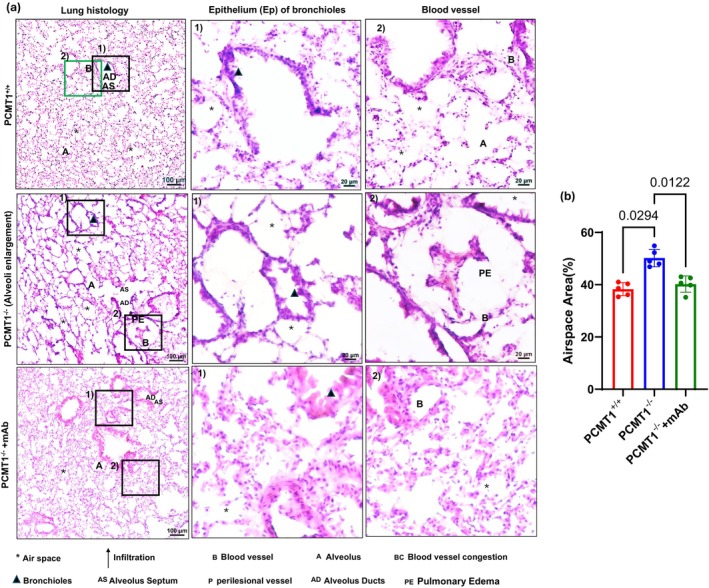
IsoDGR mAb treatment improves emphysema phenotype in lungs from Pcmt^−/−^ mice Representative H&E‐stained lung sections from 5 to 6 week‐old Pcmt1^+/+^, and Pcmt1^−/−^ mice, displaying airspace enlargement, severe pulmonary oedema, and alveolar destruction, except in Pcmt1^−/−^ mice treated with anti‐isoDGR mAb therapy. Magnified images show representative lung edema, blood vessel congestion, epithelial (Ep) swelling in bronchioles, and leukocyte infiltration in untreated Pcmt1^−/−^ animals (*n* = 9). (b) Graph represents quantification of airspace in lungs from Pcmt1^+/+^, Pcmt1^−/−^, and Pcmt1^−/−^ + mAb at 5–6 weeks. Statistical significance was assessed using one‐way ANOVA, and the results are presented as mean values with standard errors (SEM).

We next performed H&E staining to better visualise lung histology in Pcmt1^+/+^, Pcmt1^+/−^, Pcmt1^−/−^, and mAb‐treated Pcmt1^−/−^ mice. These analyses revealed enlargement of the perivascular space, blood vessel congestion, airspace distension, and increased perivascular leukocyte infiltration in Pcmt1^−/−^ mouse lungs (Figure 3). When lung phenotypes were assessed by MRI, we observed increased opacity and consolidation in Pcmt1^−/−^ and 2‐year‐old Pcmt^+/−^ mice relative to Pcmt1^+/+^ controls, consistent with significant pulmonary inflammation and edema (Figure S4). Pathological assessment also confirmed severe alveolar edema that likely contributed to the premature death of Pcmt1^−/−^ mice (10% very severe oedema, 50%–60% moderate, 30% less severe). Several previous reports have shown that reduced SpO2 levels in mice of advanced age is due to enlargement of alveoli, leading to impaired gas exchange with the circulation. Strikingly, weekly treatment with isoDGR‐specific mAb was able to limit airspace enlargement and improved lung histology in 5–6‐week‐old Pcmt1^−/−^ mice (Figure 3).

### 3.5 Positive correlation of CD68+ lung leukocytes with isoDGR‐modified extracellular matrix

Immunostaining of lung tissues revealed marked accumulation of isoDGR‐damaged proteins in the lung parenchyma and perivascular walls of Pcmt1^−/−^ mice compared with control mice (Figure S5). Intriguingly, isoDGR distribution appeared to coincide with CD68^+^ leukocyte infiltration (Figure S5A–C), suggesting that this motif may promote monocyte–macrophage recruitment to damaged ECM components. Indeed, the extent of isoDGR accumulation in lung tissue was found to be positively correlated with CD68^+^ monocyte–macrophage localisation (Figure S5D). Subsequent immunostaining revealed that isoDGR distribution also correlated with F4/80^+^ cells (Figure S6) and fibronectin in the lungs of Pcmt1^−/−^ mice (Figure S7), further suggesting that isoDGR‐modified ECM components can recruit monocyte‐macrophages to induce pulmonary inflammation. Importantly, mAb treatment reduced isoDGR‐modified protein levels in the lungs of Pcmt1^−/−^ mice, suggesting that immune‐mediated clearance of this damage motif can reduce age‐linked tissue inflammation.

### 3.6 Antibody therapy reverses age‐linked increases in isoDGR‐mediated lung inflammation

**FIGURE 4:**
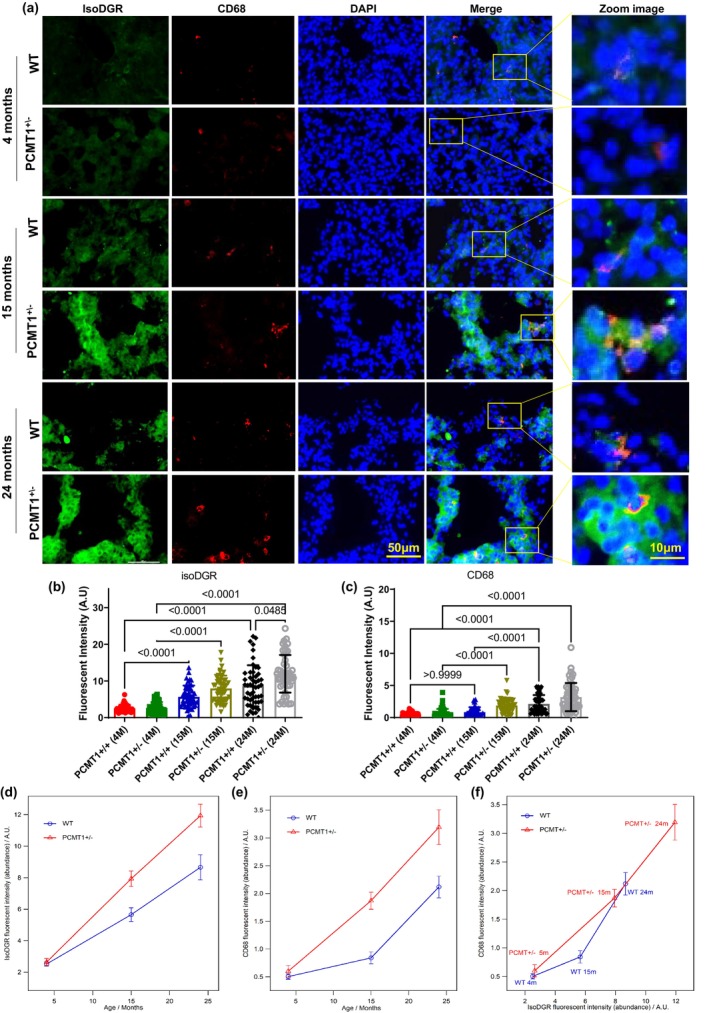
Age‐induced accumulation of isoDGR‐modified proteins correlates with CD68+ lung leukocytes in Pcmt1^+/−^ mice. (a) Representative immunostaining images showing isoDGR‐protein distribution and correlation with CD68^+^ macrophages in cryosectioned lung tissue from Pcmt1^+/+^, Pcmt1^+/−^, Pcmt1^−/−^, and mAb‐treated Pcmt1^−/−^ mice at 4, 15, or 24 months (*n* = 3). isoDGR (b) and CD68 (c) fluorescence were quantified in Image J using 50 randomized regions from 3 images of 3 independent lung sections for each genotype (graphs show average values for the same region from 3 images). (d) Plot showing age‐linked accumulation of isoDGR‐motif in both Pcmt1^+/+^ and Pcmt1^+/−^ animals. (e) Graph showing age‐linked accumulation of CD68^+^ cells in both Pcmt1^+/+^ and Pcmt1^+/−^ animals. Results indicate that CD68+ macrophage infiltration is accelerated in old animals irrespective of genotype (f), with cell frequency also being proportional to isoDGR levels. Statistical significance was determined by the Kruskal–Wallis test. Results shown are mean values ± SEM.

H&E imaging of lung pathology revealed a correlation of disease severity with increasing age of Pcmt1^+/+^ and Pcmt1^+/−^ mice, although heterozygous animals displayed more severe features at the same age (assessed at 4, 15, and 24 months, Figure S8). To confirm this apparent link between age‐induced isoDGR accumulation and lung pathology, we next determined motif expression levels and infiltration of CD68+ macrophages in lung tissues from Pcmt1^+/+^ and Pcmt1^+/−^ mice aged 4, 15, or 24 months (Figure 4a–c). Analysis by lung tissue immunostaining confirmed that isoDGR‐damaged protein levels and CD68+ cell frequency increased with age in both Pcmt1^+/+^ and Pcmt1^+/−^ mice (Figure 4d,e), and that Pcmt1^+/−^ mice displayed more extensive motif accumulation. Notably, CD68 fluorescence was found to increase in proportion with isoDGR intensity, and both features accumulated faster in lungs from older mice irrespective of genotype (Figure 4f). These results strongly suggest that aging leads to accumulation of isoDGR‐damaged lung proteins that likely recruit CD68+ macrophages to perivascular and parenchymal tissues, thus creating a local inflammatory response that drives pulmonary pathology. We therefore tested whether age‐damaged protein levels can also be reduced in the lungs of naturally aged mice treated with target‐specific immunotherapy. Indeed, we observed that injection of isoDGR‐specific mAb into 17‐month‐old mice resulted in marked immune clearance of damaged lung proteins, whereas administration of an isotype‐matched IgG control antibody did not (Figure S9).

### 3.7 Aging‐damaged isoDGR‐proteins induce both local and systemic inflammatory responses

We previously reported that isoDGR binding to macrophage surface integrins induces pro‐inflammatory cytokine release in artery walls, whereas others have observed that CD68+ alveolar macrophages contribute to severe pulmonary inflammation in emphysema / COPD (Kaku et al., 2014). Having already identified that resident macrophages co‐localise with isoDGR‐damaged lung proteins (Figure 4 and Figure S5), we next investigated whether this association could result in increased tissue inflammation. To do this, we performed reverse transcription‐quantitative polymerase chain reaction (RT‐qPCR) using total RNA extracted from the lungs of Pcmt1^+/+^, Pcmt1^+/−^, Pcmt1^−/−^, and mAb‐treated Pcmt1^−/−^ mice. Using this analysis, lungs from Pcmt1^−/−^ mice were found to display significantly higher expression of inflammatory mediators including MCP1, IL‐1α, IL‐12p40, IL‐8, CCL4, and IL‐3. Strikingly, mAb treatment of Pcmt1^−/−^ mice reduced lung tissue levels of these same inflammatory cytokines while simultaneously increasing expression of pro‐regulatory IL‐10, although this did not reach the threshold for statistical significance (Figure S10). These results suggest that isoDGR‐specific immunotherapy can promote antibody‐mediated phagocytic clearance of isoDGR‐damaged lung proteins to reduce pulmonary inflammation (Kalailingam et al., 2023). Similarly, when we quantified inflammatory cytokines in 2‐year‐old mouse lungs, we detected increased tissue levels of MCP1, TNFα, and IL‐1α in Pcmt1^+/−^ mice compared with age‐matched Pcmt1^+/+^ controls (Figure S11A), consistent with age‐linked patterns of isoDGR accumulation in these genotypes.

### 3.8 Synthetic isoDGR‐modified peptides induce pulmonary inflammation

To confirm that isoDGR‐modified proteins underlie the lung damage observed in Pcmt1^−/−^ mice, we next sought to replicate this pathology in 6‐week‐old Pcmt1^+/+^ mice (C57BL/6 background) via intranasal injection of synthetic isoDGR‐peptide. Mice were administered 100 μg isoDGR‐peptide in 50 μL PBS (or PBS‐only vehicle control), and 24 h later interstitial fluid was collected from the lungs for determination of pro‐inflammatory cytokine levels. We detected significantly higher levels of inflammatory cytokines TNFα and IL‐6 in lung fluid from isoDGR peptide‐treated mice relative to those that received only vehicle control (Figure S11B). Further analysis by immunostaining also revealed that residual isoDGR‐peptides were co‐localized with CD68^+^ immune cells in the lungs of treated mice (Figure S11C) and that the extent of CD68 infiltration was positively correlated with isoDGR accumulation (Figure S11D–F). Together, these results clearly indicate that isoDGR exerts pro‐inflammatory effects in murine lungs.

### 3.9 Transcriptomic profiling reveals TLR pathway activation in Pcmt1‐KO mouse lung

Persistent low‐grade inflammation in elderly individuals contributes to the risk of age‐linked disorders including CVD, cancer, and chronic pulmonary disease (Akgün et al., 2012). Previous studies have shown that damage‐associated molecular patterns (DAMPs) can be recognized via Toll‐like receptors (TLRs) that cross‐talk with IL‐1 signalling pathways and activate pro‐inflammatory gene expression (Alvarez & Vasquez, 2017; Cohen, 2014). To further assess the pathological role of isoDGR in vivo, we next used RNA‐seq to profile the lung tissue transcriptome of Pcmt1^+/+^, Pcmt1^*−/−*^, and mAb‐treated Pcmt1^*−/−*^ mice. We identified a total of 13,920 genes in Pcmt1^+/+^ mice and 14,094 genes in Pcmt1^*−/−*^ mice (13,611 genes were shared in common, while 443 genes were differentially expressed between genotypes) (Figure S12A). Of the genes differentially expressed in Pcmt1^*−/−*^ mice, 111 (~25%) could be restored to Pcmt1^+/+^ levels by administration of anti‐isoDGR mAb (Figure S12A,B). Gene ontology analysis revealed that these 111 genes were critically involved in transcription, translation, and various other biological roles crucial for cell structure, function, and survival (Figure S12B). When subjected to cluster analysis, we identified 30 upregulated genes in Pcmt1^*−/−*^ mice, of which 21 could be restored to normal levels by mAb treatment. Similarly, of the genes downregulated in Pcmt1^*−/−*^ mice, a majority of these were successfully normalised by mAb administration (12 of 21 genes; Figure S12C,D). These transcriptomic data were then validated by RT‐qPCR, which confirmed increased expression of FN1 and decreased levels of MT2, RGcc and PDK4 in the lungs of untreated Pcmt1^−/−^ mice (Figure S12E–H). When we focused our analyses on TLR‐mediated inflammatory pathways, we observed dysregulation of 31 key genes (21 increased, 10 decreased) in lung tissue from Pcmt1^*−/−*^ mice compared to Pcmt1^*+/+*^ mice. Similar to our previous analysis, 18 of 21 genes upregulated in Pcmt1^*−/−*^ mice were normalised upon mAb treatment (including potent effects on *TLR1*, *TLR2*, *TLR4*, *TLR6*, *Myd88*, and *IRK4*), whereas 9 of 10 downregulated genes were restored to Pcmt1^+/+^ levels by isoDGR‐specific immunotherapy.

### 3.10 Aging‐damaged isoDGR proteins activate TLR pathways

To confirm the role of isoDGR in activation of TLR pathways, we next performed RT‐qPCR on total pulmonary RNA, which revealed increased expression of TLR1, TLR4, TLR8, TIRAP, FADD, and TRAF6 in the lungs of Pcmt1^−/−^ mice (Figure S12I–N). Strikingly, motif‐specific mAb treatment was able to significantly reduce mRNA levels of these same TLRs in lung tissue from Pcmt1^−/−^ mice. Next, we studied isoDGR effects on TLR pathways using endothelial cells incubated on plates coated with native fibronectin (FN) or modified FN (isoDGR‐FN) in the presence or absence of motif‐specific mAb (or PBS‐only control). RT‐qPCR analysis revealed that HUVECs upregulated expression of TLR3, TLR4, TLR8, and ICAM when exposed to isoDGR‐FN but not native FN or PBS only (Figure S13). Addition of isoDGR‐mAb efficiently blocked TLR induction in HUVECs co‐cultured with isoDGR‐FN. Consistent with these data, Pcmt1^−/−^ knockdown in HUVECs (HUVECPcmt1^−/−^) confirmed the ability of isoDGR to trigger TLR pathway activity, which was further associated with a significant reduction in proliferation and migratory activity relative to Pcmt1^+/+^ cells (Figure S14). We then proceeded to quantify mRNA expression levels of TLR‐associated genes in Pcmt1^+/+^ and Pcmt1^+/−^ mice at age 4, 15, and 24 months. These data revealed that TLR1, TLR9, and IRK4 expression levels increased over time in Pcmt1^+/−^ mice (Figure S12O). These results are in line with previous reports that isoDGR‐modified fibronectin can activate macrophages via integrin “outside‐in” signalling, thereby triggering an ERK:AP‐1 cascade and pro‐inflammatory release of MCP1 and TNFα, (Park et al., 2021) which likely impact on TLR pathway activity (Alvarez & Vasquez, 2017; Cohen, 2014; Lucas et al., 2021).

### 3.11 IsoDGR‐specific immunotherapy reduces Pcmt1^−/−^ lung pathology as determined by single‐cell RNA sequencing

**FIGURE 5:**
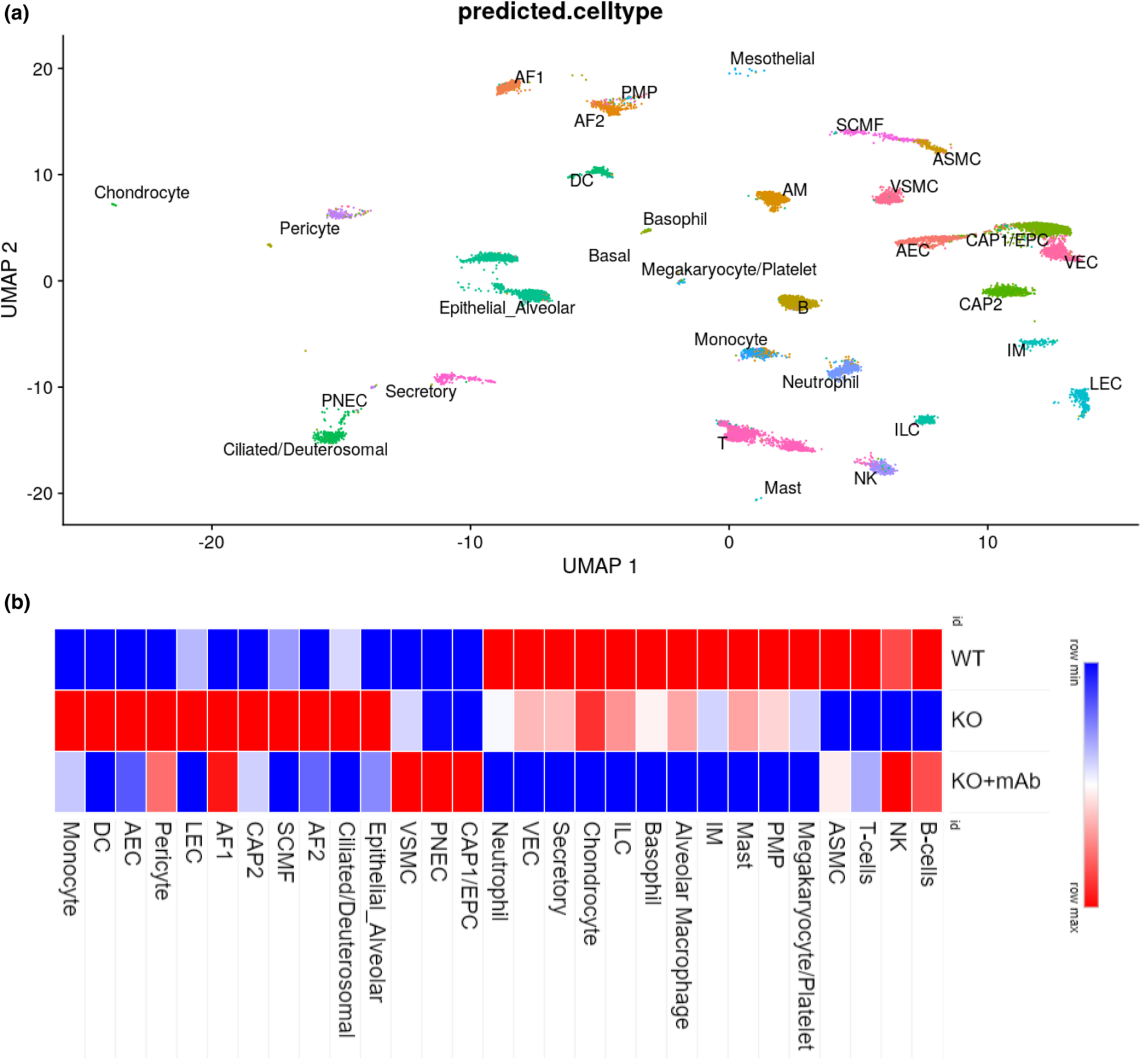
Lung cell landscape of Pcmt1^+/+^, Pcmt1^−/−^, and mAb‐treated Pcmt1^−/−^ mice (a) UMAP representation of the lung scRNA‐seq results. Cell clusters were annotated with LungMap‐defined cell types using the Azimuth algorithm. A total of 29 different lineages were identified in murine lung. (b) Heatmap showing clusters and relative abundance of 29 lung cell types in the WT, Pcmt1 KO, and mAb‐treated Pcmt1 KO mice.

To gain a deeper understanding of isoDGR‐induced pulmonary disorder in Pcmt1^−/−^ mice, we next conducted single‐cell RNA sequencing of lung tissues from Pcmt1^+/+^, Pcmt1^−/−^, and mAb‐treated Pcmt1^−/−^ mice at 6 weeks old. We identified 29 distinct cell clusters, including lung‐specific epithelial and structural cells, vascular and endothelial cells, as well as various leukocyte subsets (Figure 5a). Among these clusters, B cells, T cells, and natural killer cells were markedly decreased in lung tissues from Pcmt1^−/−^ mice, whereas monocytes and dendritic cells were significantly increased, perhaps reflecting active infiltration by phagocytic cells and subsequent tissue inflammation. Imbalance of myeloid and lymphoid populations in the lung may be sufficient to enhance tissue injury and infection risk, while also inhibiting repair and resolution of inflammation (Melms et al., 2021). Our finding that cellular composition of the lungs is altered in knockout mice suggests a potential role for these cell types in the aging lung phenotype.

The observed decrease in number of B cells and NK cells may disrupt normal immune surveillance, thus increasing susceptibility to infections and inflammation. Changes in abundance of epithelial alveolar AF2 cells and Ciliated/deuterostome cells may also disturb lung physiology and contribute to the age‐related decline in respiratory function. Additionally, SCMF (submucosal fibroblasts), CAP2 (capillary endothelial cells), LEC (lymphatic endothelial cells), and AEC (alveolar epithelial cells) are implicated in various aspects of lung structure and function. Dysregulation of these cell types could contribute to age‐related lung diseases such as fibrosis, pulmonary edema, and impaired immune function. Dendritic cells (DCs) and monocytes play crucial roles in antigen presentation, immune surveillance, and control of inflammation. Alterations in their abundance and function with age may impact the lung's ability to mount effective immune responses and resolve inflammation, potentially leading to chronic lung diseases and impaired tissue repair. Conversely, the reduction in T cells, ASMC (airway smooth muscle cells), VSMC (vascular smooth muscle cells), PNEC (pulmonary neuroendocrine cells), and CAPI/EPC (club cells/Epithelial progenitor cells) observed in knockout mouse lungs may reflect a decline in tissue repair and regenerative capacity, as well as compromised immune surveillance against pathogens and malignant cells. The reversal of these cellular features of knockout mouse lungs following mAb treatments suggests that targeting specific populations can ameliorate age‐related changes in lung phenotype. By restoring the balance of immune, epithelial, and structural cells, mAb treatment may promote lung health and function in aging individuals. Further research is warranted to elucidate the mechanisms underlying the therapeutic effects of the mAb and to explore its potential as novel interventions for age‐related lung diseases.

Strikingly, mAb treatment restored both myeloid and lymphoid cell frequencies to the levels observed in Pcmt1^+/+^ mice (Figure 5b). Our scRNA‐seq data also revealed that Pcmt1^−/−^ mouse lungs exhibit increased numbers of pericytes, which are specialized cells that surround capillaries and regulate blood flow to downstream tissues (Davis & Attwell, 2023; Peng et al., 2013). High frequencies of lung pericytes in Pcmt1^−/−^ mice might indicate suboptimal pulmonary circulation and endothelial barrier function, potentially related to the compromised gas exchange and increased lung permeability observed previously (Figure 2). Together, these findings confirm that isoDGR accumulation in Pcmt1^−/−^ mouse lungs are associated with several pathophysiological defects, including blood vessel barrier issues, impaired gas exchange, and an altered immune profile that can be corrected by mAb treatment.

### 3.12 Anti‐isoDGR mAb treatment restores ribosomal protein expression in Pcmt1^−/−^ mouse lung

Pcmt1^−/−^ mice displayed significantly reduced weight compared to their control littermates. Among the genes found to be dysregulated upon scRNA‐seq analysis of Pcmt1^−/−^ mouse lung, we identified a subset of proteins associated with ribosomal machinery that were significantly decreased relative to littermate controls (including RPS35, RPS36, RPS38, RPS21, and RPS28; Figure S15). Administration of anti‐isoDGR mAb treatment appeared to increase expression of these ribosomal genes, so we next sought to validate the scRNA‐seq data by RT‐qPCR analysis of mouse lung tissues. This analysis confirmed that ribosomal gene expression was decreased in Pcmt1^−/−^ mouse lungs and could be partially restored by mAb treatment. Further studies will be necessary to fully elucidate how isoDGR‐modified proteins impact the ribosomal machinery in vivo, since this finding may be directly linked with the reduced body weight and short lifespan of Pcmt1^−/−^ mice. Nonetheless, mAb‐induced clearance of isoDGR‐modified lung proteins was associated with marked weight gain and lifespan extension in Pcmt1^−/−^ mice, perhaps in part due to restoration of protein synthetic functions.

### 3.13 IsoDGR impairs pulmonary ETC and mitochondrial functions

**FIGURE 6:**
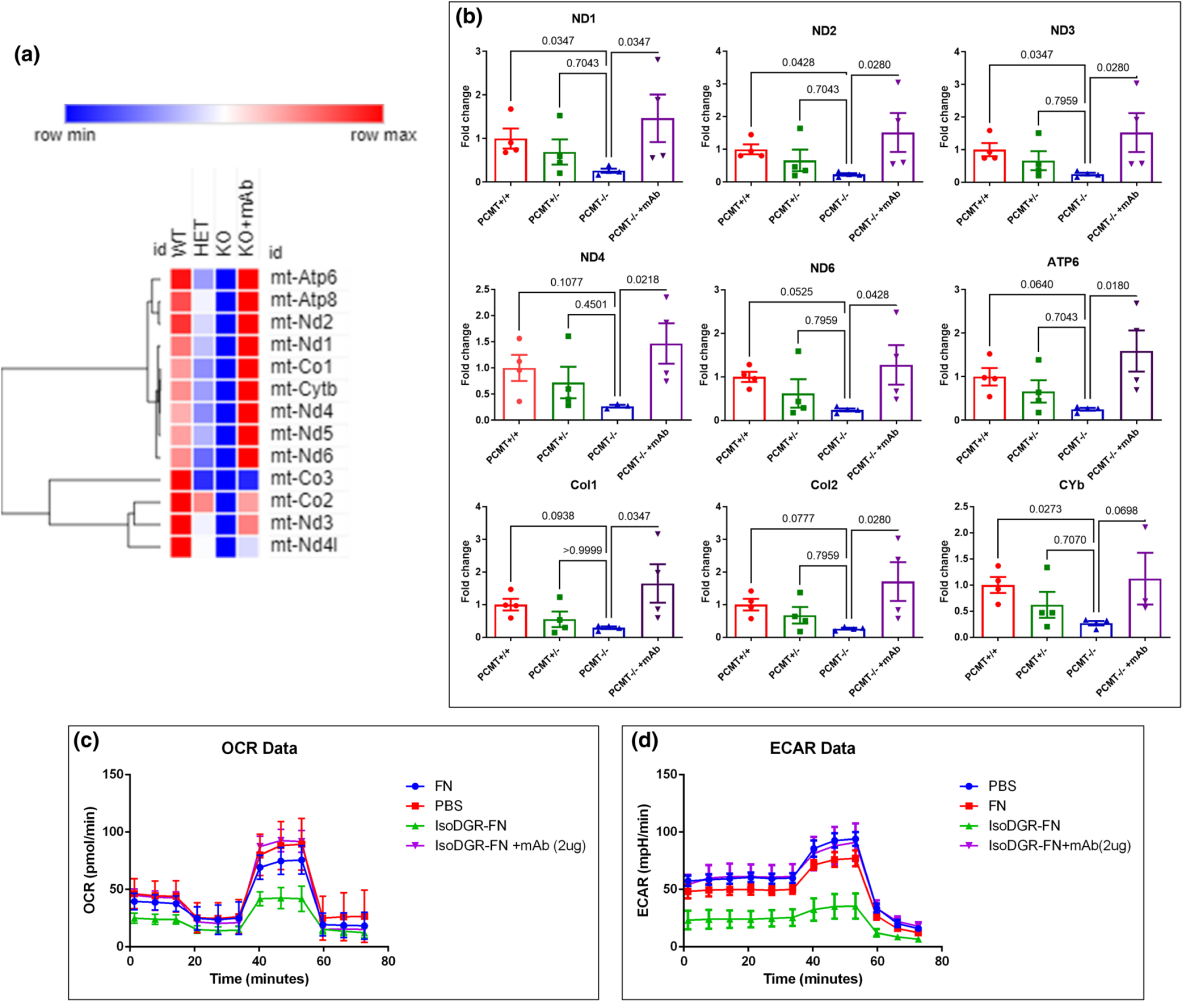
IsoDGR impairs ETC and mitochondrial functions in murine lung. (a) Heatmap of transcriptomic data showing that substantial down‐regulation of mtDNA expression in lung tissue from Pcmt1^−/−^ mice can be restored by anti‐isoDGR mAb treatment. (b) Graph showing quantitative PCR analysis of mtDNA and ETC‐related gene expression in the lungs of Pcmt1^+/+^, Pcmt^+/−^, Pcmt^−/−^, and mAb‐treated Pcmt^−/−^ mice aged 6 weeks. Data were normalised to GAPDH expression level. Statistical significance was determined by the Kruskal–Wallis test. Results shown are mean values ± SE. (*n* = 4) (c, d) HULEC‐5a were seeded into Seahorse Bioscience microplates for assessment of mitochondrial function‐associated OCR (c) and ECAR (d) using a XF96 extracellular flux analyser (*n* = 12–16). Statistical significance was assessed using one‐way ANOVA, and the results are presented as mean values with standard errors (SEM).

Mitochondrial dysfunction is a hallmark of aging and has been observed in both acute and chronic lung diseases (Cloonan et al., 2020). Several reports have shown that ageing modulates mitochondrial DNA structure and functions, leading to dysregulation of energy metabolism (reduced ETC capacity and decreased ATP production) as well as enhanced ROS production (Barnes, 2020, 2022; Wiegman et al., 2015). Our RNA sequencing data indicated that ETC‐linked gene expression was substantially reduced in the lungs of Pcmt1^−/−^ compared to control mice (Figure 6a). Validation of the RNA sequencing results by RT‐qPCR also confirmed that lung tissue from Pcmt1^−/−^ mice displayed reduced expression of numerous ETC‐associated genes (including ND1, ND2, ND3, ND4, NDL4, ND5, ND6, ATP6, Col1, Col2, and Cytb). Crucially, isoDGR‐mAb administration to Pcmt1^−/−^ mice was able to restore normal expression levels of these dysregulated mtDNA genes, suggesting re‐establishment of lung mitochondrial functions following treatment (Figure 6b). Since ETC defects are known to result in electron leakage, ROS generation, and inefficient respiration, we next performed Seahorse and CellROX assays to determine whether isoDGR effects on mitochondria also impact energy metabolism. To do this, we seeded human lung endothelial cells (HULEC‐5a) into PBS only, native FN, or isoDGR‐modified FN, in the presence or absence of anti‐isoDGR mAb. These experiments revealed that HULEC‐5a cells exposed to isoDGR‐FN displayed reduced oxygen consumption rate (OCR) and extracellular acidification rate (ECAR) relative to control cells (PBS only). However, isoDGR‐specific mAb treatment was able to restore OCR and ECAR to normal levels in HULEC‐5a cells (Figure 6c,d). Similarly, HULEC‐5a cells generated significantly higher levels of ROS when cultured in the presence of isoDGR‐FN, but this was significantly reduced by co‐incubation with isoDGR‐specific mAb (Figure S16). Several reports have shown that ECM stiffness can trigger abnormal integrin signaling and mitochondrial dysfunction, thereby contributing to the development of disorders such as COPD, lung fibrosis, and emphysema (Burgstaller et al., 2017; Guo, He, et al., 2022; Liu et al., 2022; Nho et al., 2022). These results are consistent with our present finding that isoDGR‐modified ECM proteins promote age‐linked mitochondrial dysfunction in Pcmt1^−/−^ mouse lung, whereas targeted antibody therapy can induce immune clearance of this damage motif and restore normal metabolic activity.

### 3.14 IsoDGR promotes senescence of lung parenchymal and endothelial cells

**FIGURE 7:**
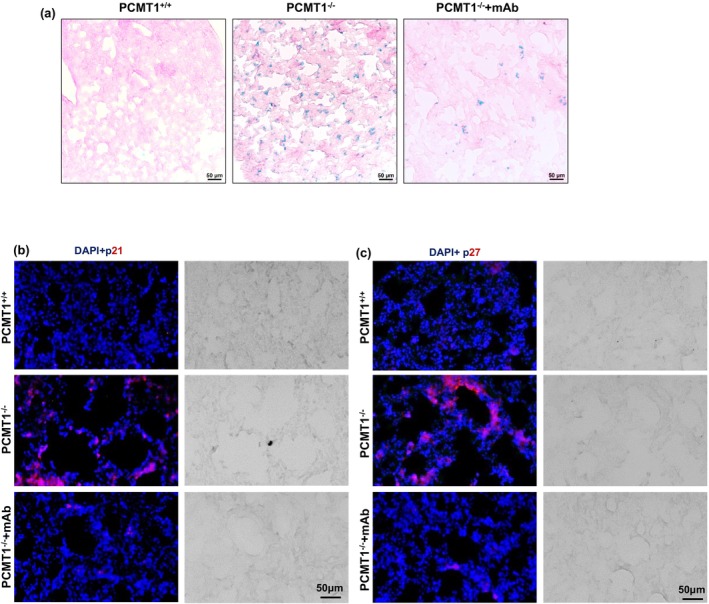
IsoDGR promotes senescence of lung parenchymal and EC cells in Pcmt1^−/−^ mice. Representative immunostaining images showing β‐gal (a), P21 (b), and P27 (c) levels in lung tissue from the respective genotypes/treatment conditions (*n* = 5).

ROS induced by smoking, environmental, and other oxidative stresses are known to drive inflammation and cellular senescence in lung tissues, leading to chronic lung pathologies (Childs et al., 2015; Cloonan et al., 2020; Parimon et al., 2021). Having observing that cellular ROS levels increase with isoDGR accumulation in lung tissue from Pcmt1−/− mice, we next proceeded to investigate whether isoDGR can also promote cellular senescence and apoptosis. To test this hypothesis, we performed β‐galactosidase (β‐gal) and TUNEL staining of lung tissue obtained from Pcmt1^+/+^, Pcmt1^−/−^, and mAb‐treated Pcmt1^−/−^ mice. We observed significantly increased β‐gal levels (Figure 7) and apoptotic cell numbers (Figure S17) in lung parenchyma and endothelium from Pcmt1^−/−^ mice, whereas mAb treatment substantially reduced these pathological features. Consistent with the β‐gal and Tunel staining, we also observed increased expression of P21, P27, and caspase‐3 in lung tissue from Pcmt1^−/−^ mice (Figure 7 and Figure S17), as well as elevated frequencies of apoptotic lung parenchymal and endothelial cells in 2‐year‐old Pcmt1^+/+^ and Pcmt1^+/−^ mice relative to young mice (Figure S17C). These data further indicated that relative levels of isoDGR accumulation over the course of natural aging directly correlate with cellular dysfunction and tissue degradation in vivo. Indeed, our results are consistent with reports that protease‐mediated clearance of age‐damaged proteins can reduce cellular senescence and apoptosis to improve tissue function (Saez & Vilchez, 2014). Together, these findings clearly indicate that pulmonary accumulation of isoDGR‐modified proteins leads to increased cellular senescence and apoptosis, whereas motif‐specific mAb can promote immune clearance of damaged proteins and reduce lung pathology.

### 3.15 Anti‐isoDGR mAb treatment improves pulmonary function and promotes healthy aging

We continuously evaluate the therapeutic efficacy of isoDGR‐mAb in promoting healthy aging. IsoDGR‐mAb was administered to naturally aged mice at 18–20 months of age, and their activities were monitored in a Digital Ventilated Cages (DVC) system. This system allowed for continuous assessment of physical activity and treatment effects within their home cages (Figure S18A). Additionally, voluntary running wheels (VRW) were introduced into the DVC to assess the therapy's impact on learning and physical activity. Remarkably, old mice (20–26 months) previously treated with isoDGR‐mAb quickly learned to use the VRW and exhibited significantly enhanced running performance compared to age‐matched control groups (Figure S18B), indicating the health‐promoting effects of isoDGR‐mAb. We also monitored the mice in metabolic cages to evaluate respiratory functions. The results demonstrated that mAb‐treated old mice showed significant improvements in oxygen intake and respiratory quotient compared to control mice (Figure S18C,D). Furthermore, experiments were conducted to test the specificity of isoDGR‐peptides in inducing pulmonary inflammation compared to the native RGD‐integrin ligand. The results showed that only isoDGR‐peptides induced TNFα and IL‐6 expression (Figure S18E,F).

## 4 DISCUSSION

Aging is the primary risk factor for many chronic diseases that increase risk of early mortality. As the global population becomes more elderly, current projections suggest this will drive an exponential increase in worldwide burden of age‐linked diseases and associated healthcare costs (Nikolich‐Zugich et al., 2016). Consequently, there is an urgent need to better understand the complex processes that underpin human aging, so that we can begin to develop methods of extending healthy lifespan in elderly cohorts. It has long been recognized that the gradual accumulation of molecular damage over time leads to the deterioration of tissue function as individuals age (Gallart‐Palau et al., 2019). In particular, age‐linked DPMs have been shown to alter protein structure and bioactivity in vivo, leading to increased risk of several major diseases (Gallart‐Palau et al., 2019; Santos & Lindner, 2017). Given that molecular damage like isoDGR formation arise spontaneously, these motifs have long been regarded as untreatable features of disease, since conventional therapeutic approaches targeting genes or enzymes prove ineffective in this domain. However, as demonstrated in this study, an antibody directed at the isoDGR motif can facilitate immune clearance of damaged isoDGR proteins in vivo, offering a promising avenue for addressing age‐related lung pathology.

The ECM is a non‐cellular component of all tissues and organs that critically supports many key biochemical and biomechanical processes. ECM proteins exhibit slower turnover than cellular proteins throughout the human body and are thus more susceptible to accumulation of age‐associated DPMs. While the effects of age‐related molecular damage can be seen in all organs, some tissues may be more seriously impacted by DPMs than others. In particular, ECM‐rich organs including the lung and blood vessels are likely to be highly susceptible to DPM accumulation, leading to time‐dependent decline in critical functions including barrier maintenance, gas exchange, and delivery of oxygen/nutrients to distant body tissues.

The lung ECM is indispensable for normal organ function, consisting of a complex array of fibrous proteins (collagen, elastin), glycoproteins (fibronectin, laminin), glycosaminoglycans (heparin, hyaluronic acid), and proteoglycans (perlecan, versican) (Hackett & Osei, 2021). Consequently, dysregulation of the ECM is now recognized as a hallmark of lung aging (Hackett & Osei, 2021). These ECM proteins are rich in NGR sequence motifs prone to deamidation, resulting in the formation of integrin‐binding isoDGR motifs that enhance leukocyte adhesion (Dutta et al., 2017; Park et al., 2021). Moreover, isoDGR structurally resembles the RGD motif that mediates binding to the integrin alpha v family, implying a potential role for this axis in the pathogenesis of chronic lung diseases such as fibrosis and emphysema (Henderson et al., 2013, 2020; Morris et al., 2003; Munger et al., 1999; Slack et al., 2022).

Aligned with these findings, our study demonstrates that age‐linked accumulation of isoDGR can trigger pathological features characteristic of major lung diseases, including pulmonary inflammation, edema, hypoxemia, vascular congestion, coagulopathy, mitochondrial dysfunction, oxidative stress, and cellular senescence. Intriguingly, while isoDGR accumulates throughout the body tissues of Pcmt1^−/−^ mice, the severity of motif‐induced lung pathology shows a strong correlation with overall mortality, highlighting pulmonary dysfunction as a crucial determinant of lifespan in this model. Importantly, our results suggest that isoDGR‐specific mAb therapy can promote immune clearance of age‐related isoDGR damage and extend the lifespan of treated mice (Kalailingam et al., 2023). It is important to consider the possibility that immune responses to this mode of treatment may arise with prolonged therapy, although the initial results presented here are highly promising. Indeed, since isoDGR accumulates throughout the entire body, isoDGR‐mAb may have broader therapeutic potential beyond lung disease alone. Therefore, this immunotherapeutic approach holds promise for clinical application in mitigating age‐related disease burden and maintaining health in elderly populations.

## AUTHOR CONTRIBUTIONS

K.P., N.M., and S.K.S. designed the project. K.P., S.C.N., R.I., A.N., K.M., B.S.L., B.S., R.M., S.T.B. performed the experiments and data analysis; P.K. and S.K.S. drafted the manuscript; E.S.C., V.A.F., R.E.K.M., J.L., P.K., E.L.T., K.L.L., I.H.S., Y.G.G., A.M.R., R.N.K., C.C., C.B., and D.K. provided resources/equipment; R.E.K.M., J.L., P.K., E.L.T., Y.G.G., K.L.L., C.C., D.K., N.M., and S.K.S contributed grants/reagents/materials/analysis tools. S.K.S. conceived and supervised the project. All authors revised the manuscript.

## CONFLICT OF INTEREST STATEMENT

None declared.

## Supporting information


Appendix S1.

## Data Availability

RNA‐seq data presented in this study are deposited in GEO‐NCBI (GSE224434). All other data are included in the main text or supplemental materials.
