# Supplementary material for: Immunotherapeutic targeting of aging‐associated isoDGR motif in chronic lung inflammation
Source: Aging Cell. 2025 Jan 5;24(4):e14425. doi: 10.1111/acel.14425 (PMC11984686; doi:10.1111/acel.14425)
Supplement: Supplementary file 1 — Appendix S1. [file ACEL-24-e14425-s001.docx]

**Supplemantry Data**

**
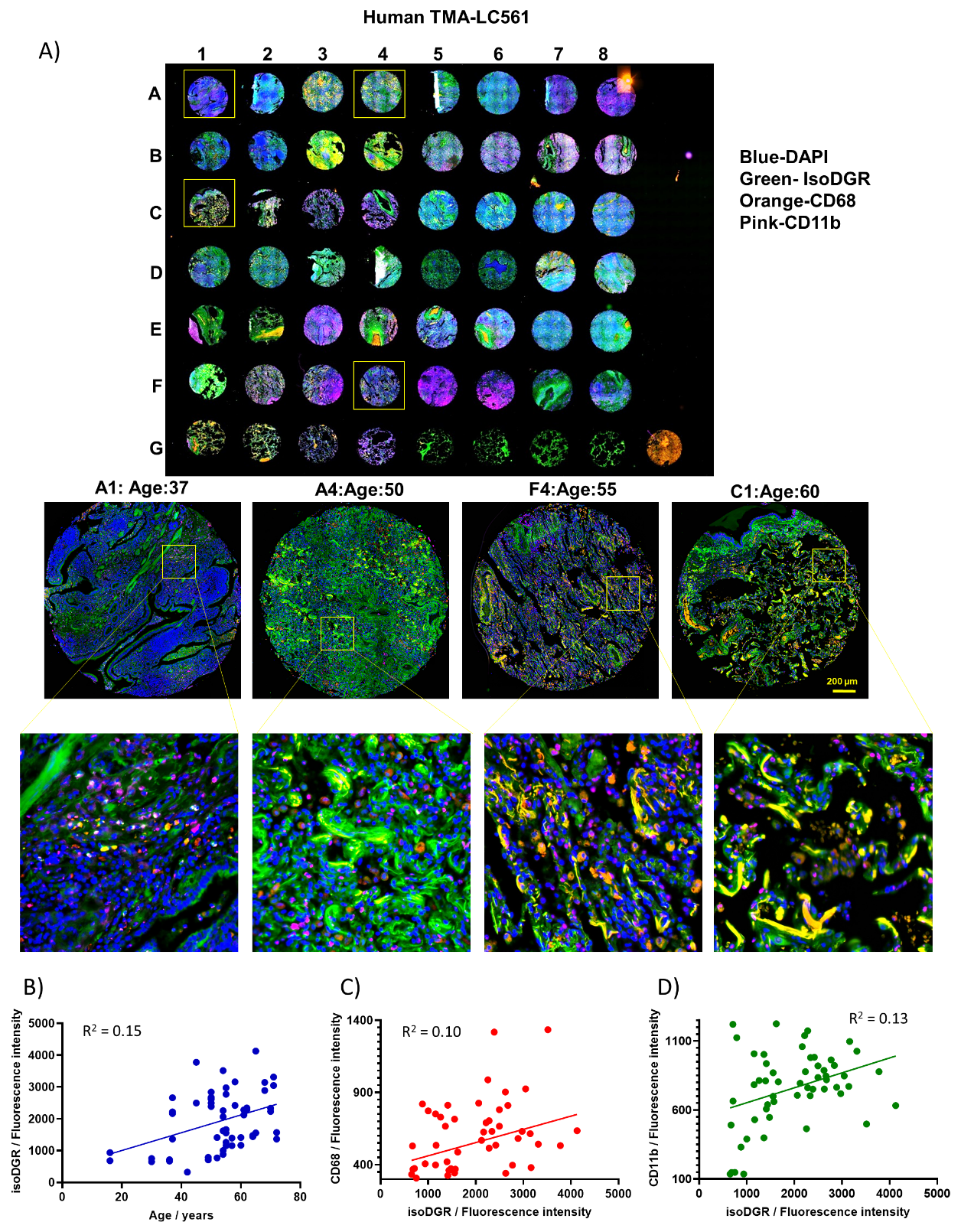
**

***Figure S1: Age-induced accumulation of isoDGR-modified proteins correlates with CD68+ and CD11b+ cells in human lung tissue.*** *(A) Representative immunostaining images showing isoDGR-protein distribution and correlation with CD68+ and CD11b+ immune cells in 56 sections of pulmonary interstitial fibrosis tissues, alongside 2 cases each of cancer-adjacent lung tissues and normal lung tissues of varying age. Representatives zoomed images are shown; A1: Age 37, A4: Age50, F4 Age 55, C1: Age 60. (B) IsoDGR level was positively correlated with age (linear regression slope 26.20). (C) CD68 level was positively correlated with isoDGR level. (D) CD11b level was positively correlated with isoDGR level.*

**
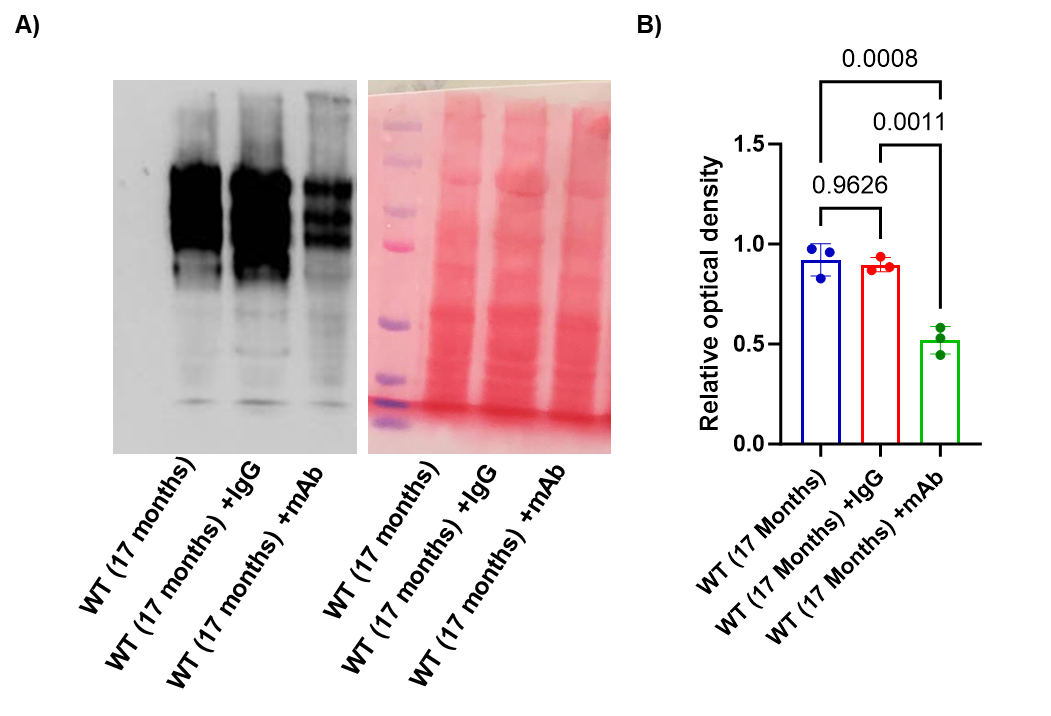
**

**Figure S2 *Immune clearance of isoDGR-damaged proteins in lung tissues of naturally-aged mice***

***(A)*** *Lung protein lysates from 17 months old WT mice, 17 months old WT mice treated with isotype IgG antibody, and 17 months old WT mice treated with isoDGR-specific mAb subjected to western blot using isoDGR-specific mAb. Protein loading was visualized by Ponceau S.(n=5)* ***(B)*** *Graph showing quantification of isoDGR-damaged protein levels in the lungs of these mice (n=5) assessed at 17 months weeks. Statistical significance was assessed using one-way ANOVA. Results are shown as mean ± SEM.*


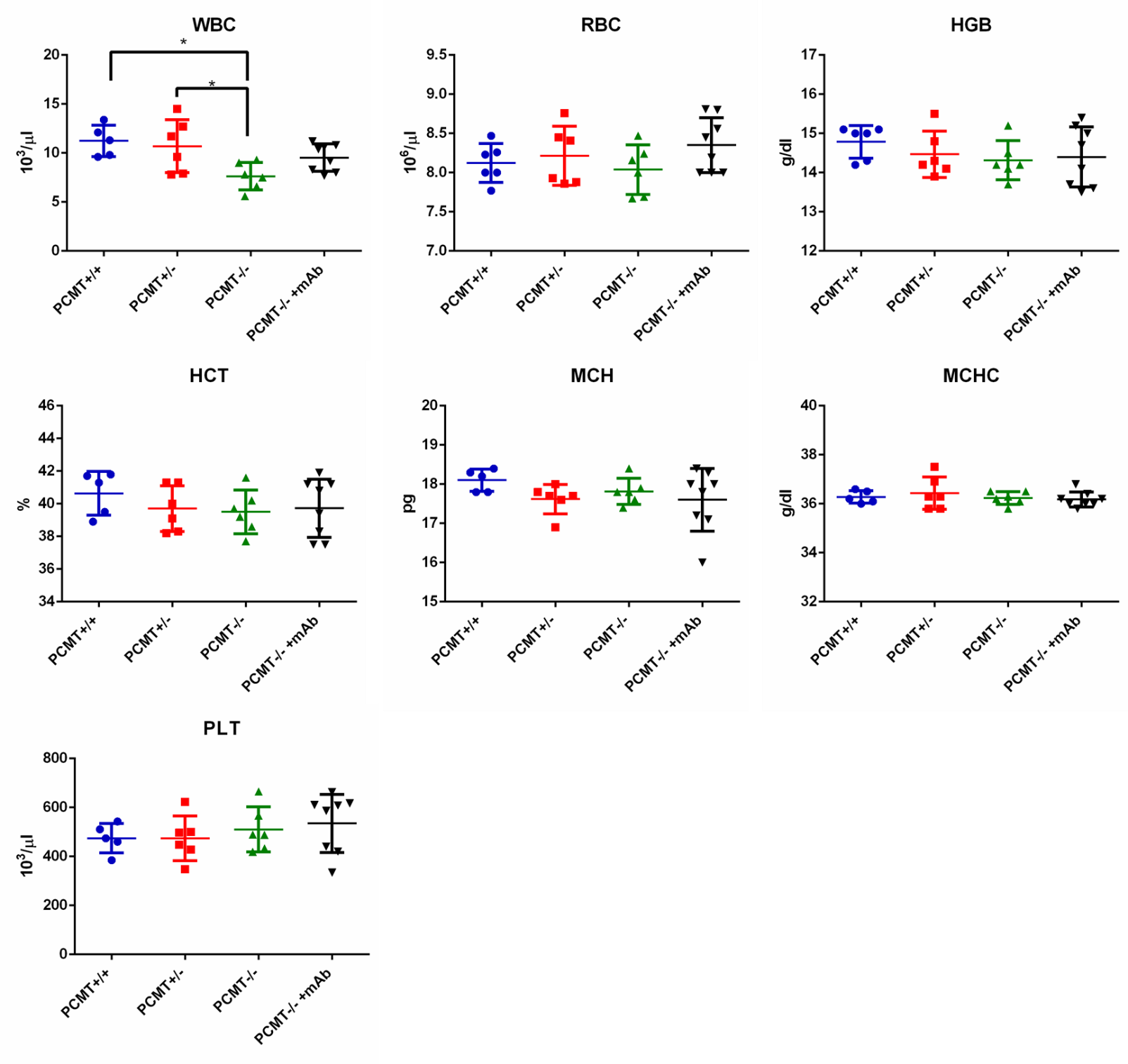


***Figure S3: Complete blood count is not altered in Pcmt1^-/-^ mice***

*Total white blood cell (WBC) count was reduced in Pcmt1^-/-^ mice, but red blood cells (RBC), haemoglobin level, oxygen transporters, and platelet numbers were not significantly altered relative to Pcmt1^+/+^ mice. Statistical significance was assessed using one-way ANOVA. Data are shown as mean ± SEM (*p < 0.05) (n=6-8).*


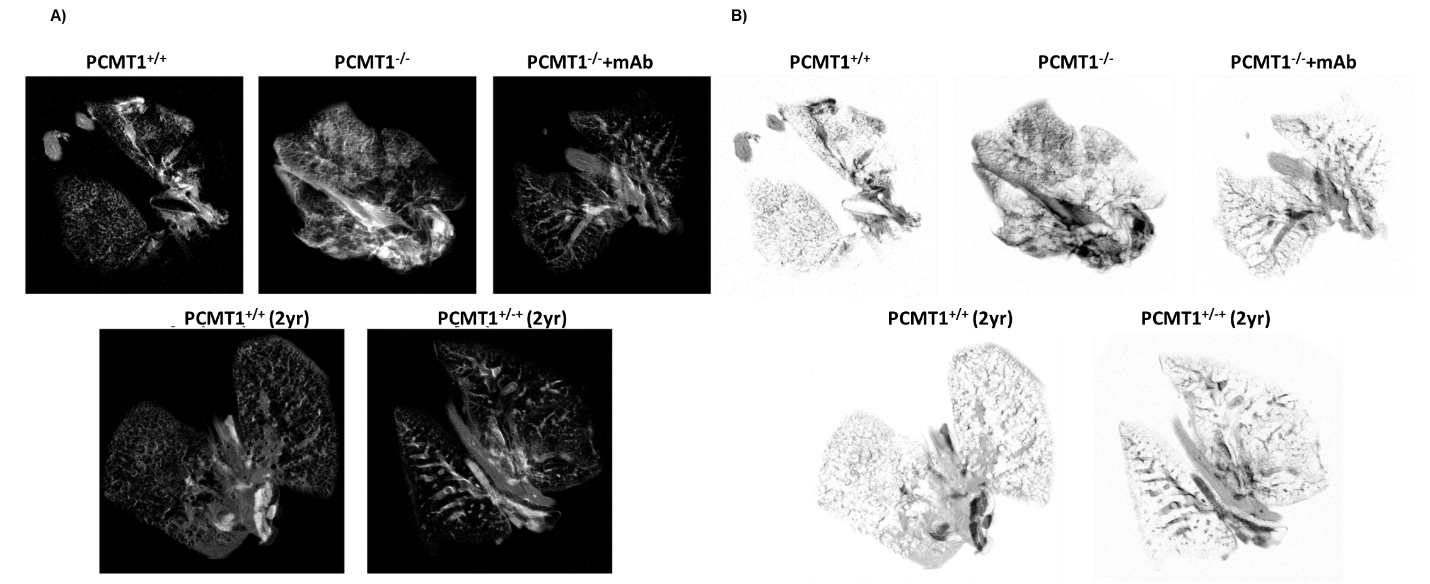


***Figure S4. MRI images of Pcmt1^+/+^, Pcmt1^+/-^, Pcmt1^-/-^, and mAb-treated Pcmt1^-/-^ lungs***

*Lung MRI representative contrast* ***(A)*** *and inverted* ***(B)*** *images for all genotypes assessed at age 5-6 weeks and 2 years (n=3).*


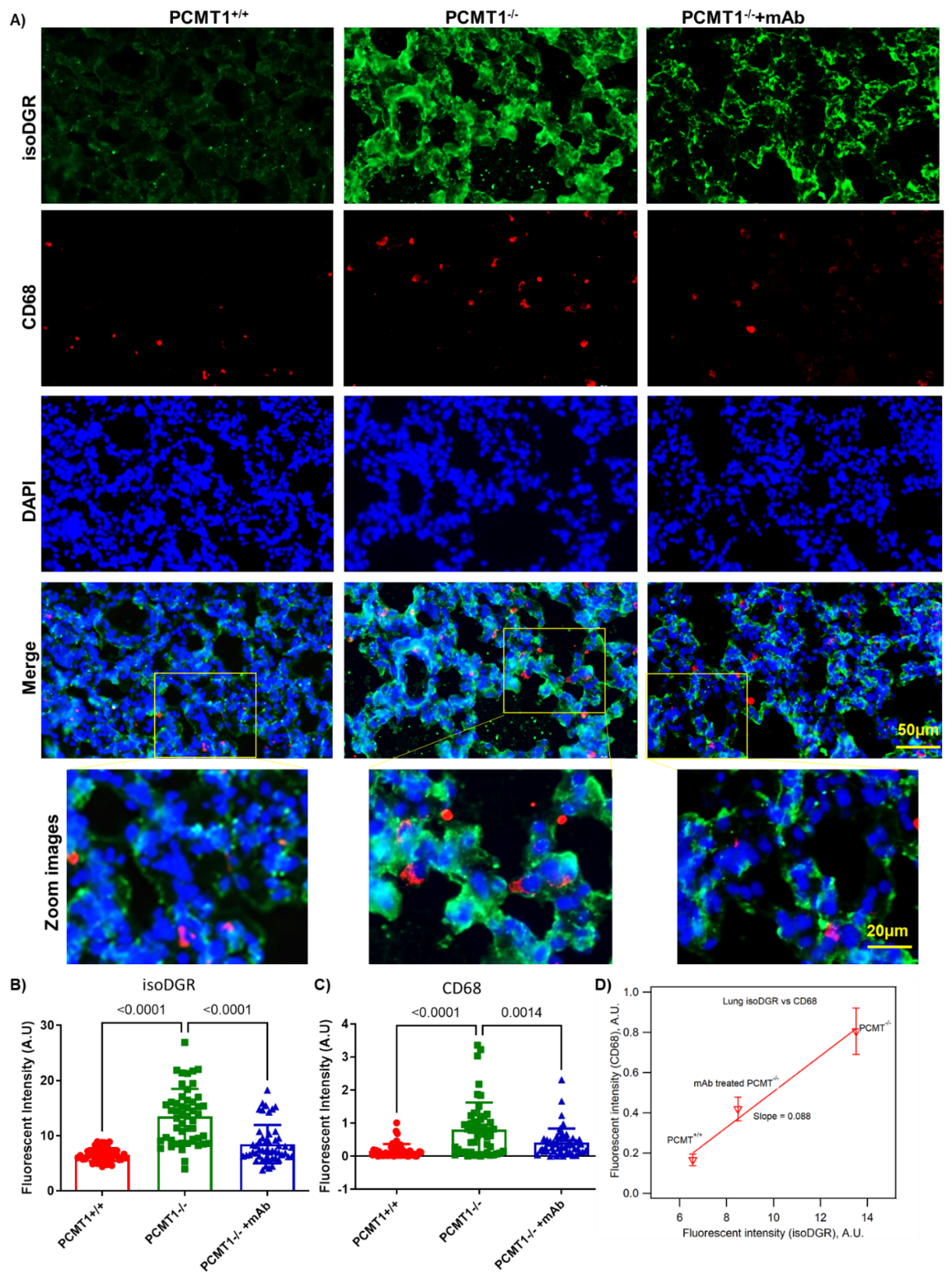


***Figure S5: IsoDGR accumulation and positive correlation with CD68+ cell infiltration of Pcmt1^-/-^ mouse lung A)*** *Representative immunostaining of isoDGR-damaged protein distribution and correlation with CD68^+^ macrophages in cryosectioned lung tissue from Pcmt1^+/+^, Pcmt1^-/-^, and mAb-treated Pcmt1^-/-^ mice at age 5-6 weeks (n=5). IsoDGR* ***(B)*** *and CD68* ***(C)*** *fluorescence were quantified in Image J using 50 randomized regions in 5 lung images from 5 individual mice for each genotype (graphs show mean values for the same region from 5 images).* ***(D)*** *Fluorescence intensity of CD68 was proportional to isoDGR intensity, indicating that motif distribution was associated with macrophage infiltration. Statistical significance was determined by Kruskal-Wallis test. Results are shown as mean values ± SEM.*

*
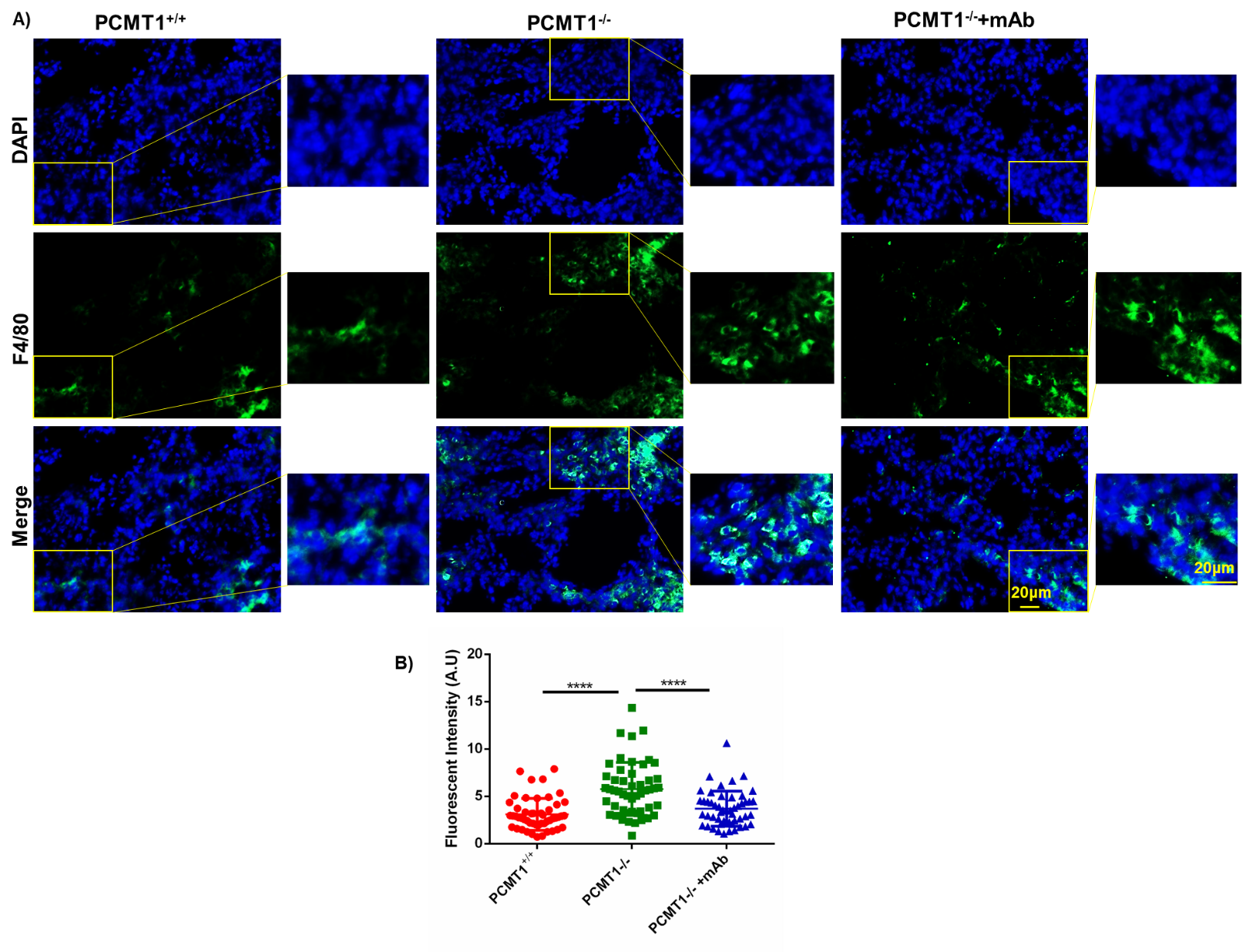
*

***Figure S6: Increased F4/80+ cell infiltration of*** *Pcmt1^-/-^* ***mouse lung***

*A) Representative immunostaining of F4/80+ macrophages in cryosectioned lung tissue from Pcmt1^+/+^, Pcmt1^-/-^, and mAb-treated Pcmt1^-/-^ mice at age 5-6 weeks (n=5-7). (B) Graph represents fluorescence intensity of F4/80+ in cryosectioned lung tissue from Pcmt1^+/+^, Pcmt1^-/-^, and mAb-treated Pcmt1^-/-^ mice. Statistical significance was assessed using one-way ANOVA. Results are shown as mean values ± SEM (**** p<0.001).*

**
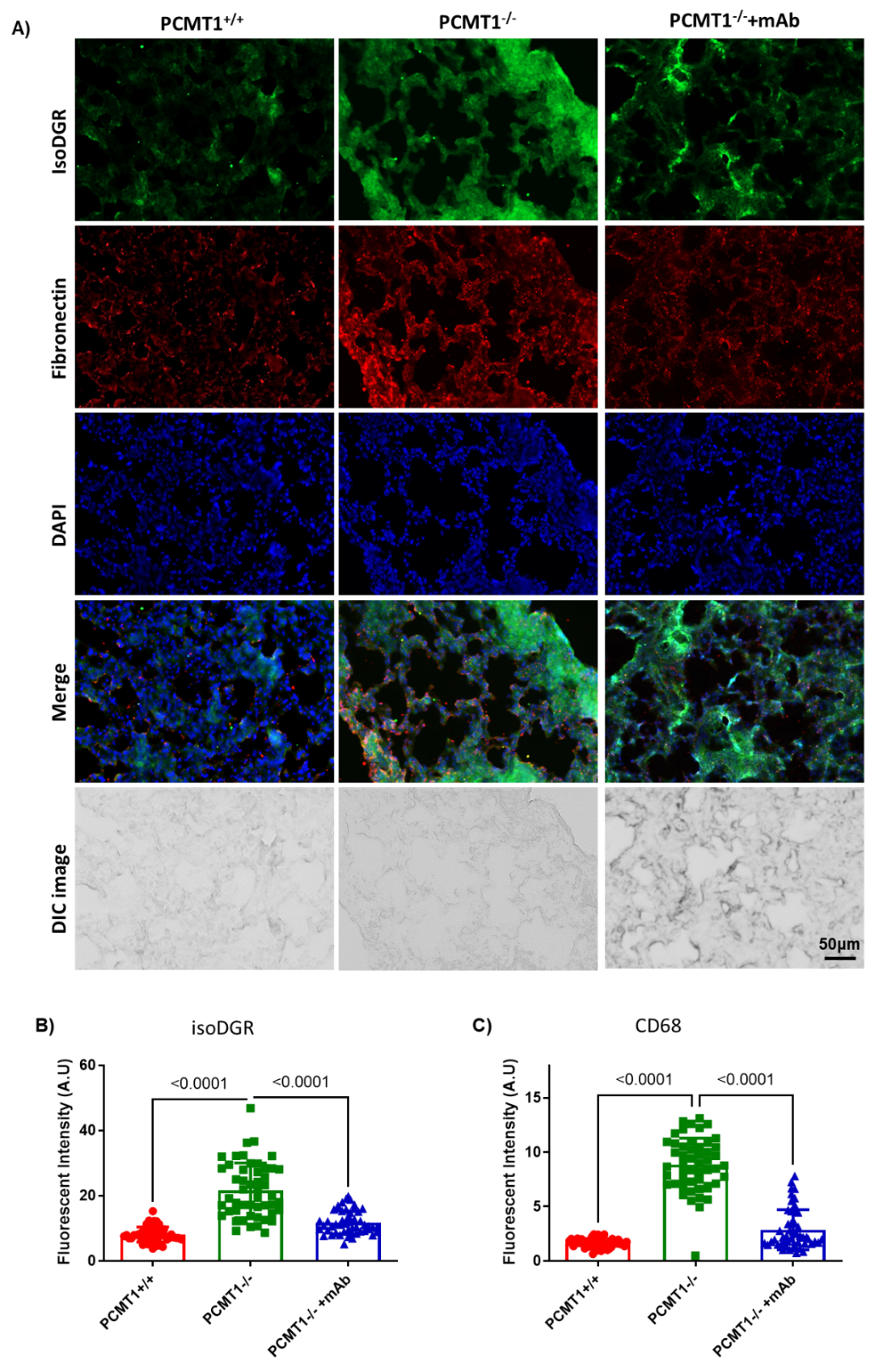
**

***Figure S7: IsoDGR accumulation and correlation with fibronectin in Pcmt1^-/-^ lung tissue***

***A)*** *Representative immunostaining of isoDGR protein distribution and correlation with fibronectin in cryosectioned lung tissue from Pcmt1^+/+^, Pcmt1^-/-^, and mAb-treated Pcmt1^-/-^ mice at age 5-6 weeks (n=3). IsoDGR* ***(B)*** *and fibronectin* ***(C)*** *were quantified in Image J using 50 randomized regions in 5 images from 5 independent lung sections for each genotype (graphs display mean values for the same region from 5 images). Statistical significance was assessed using one-way ANOVA. Results are mean ± SEM.*

*
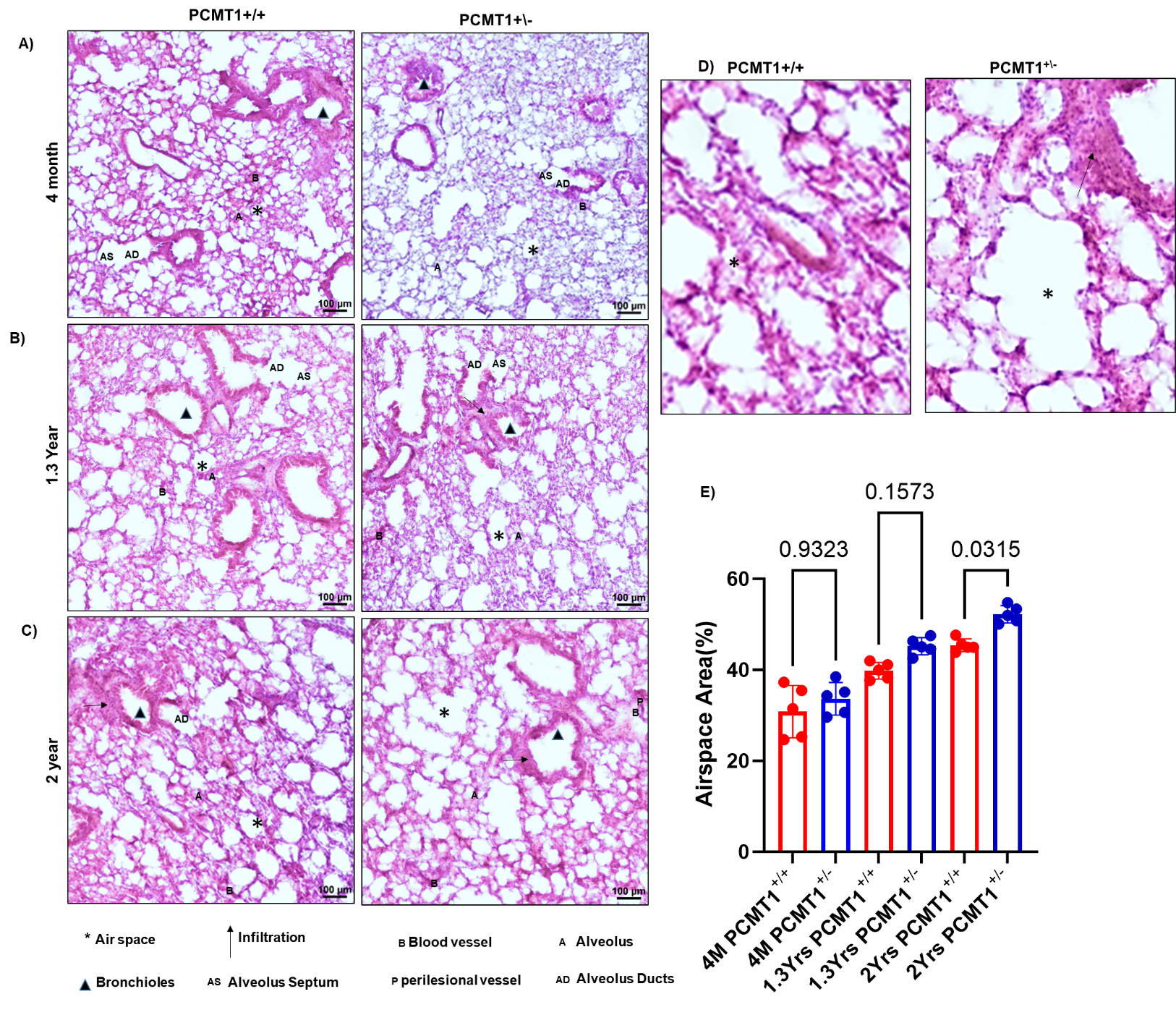
*

***Figure S8: Age-dependent airspace enlargement in lungs from Pcmt1^+/-^ mice***

*Representative images of H&E-stained lung sections from Pcmt1^+/+^ and Pcmt1^+/-^ mice assessed at 4 months (****A)****, 15 months* ***(B),*** *or 24 months* ***(C)****. Magnified H&E images show lungs from Pcmt1^+/+^ or Pcmt1^+/-^ mice with air space enlargement and immune cell infiltration* ***(D)****.* ***(E)*** *Graph represents quantification of airspace in lungs from Pcmt1^+/+^ and Pcmt1^+/-^ at 4, 15 and 24 months. Statistical significance was assessed using one-way ANOVA. Results are mean ± SEM.*


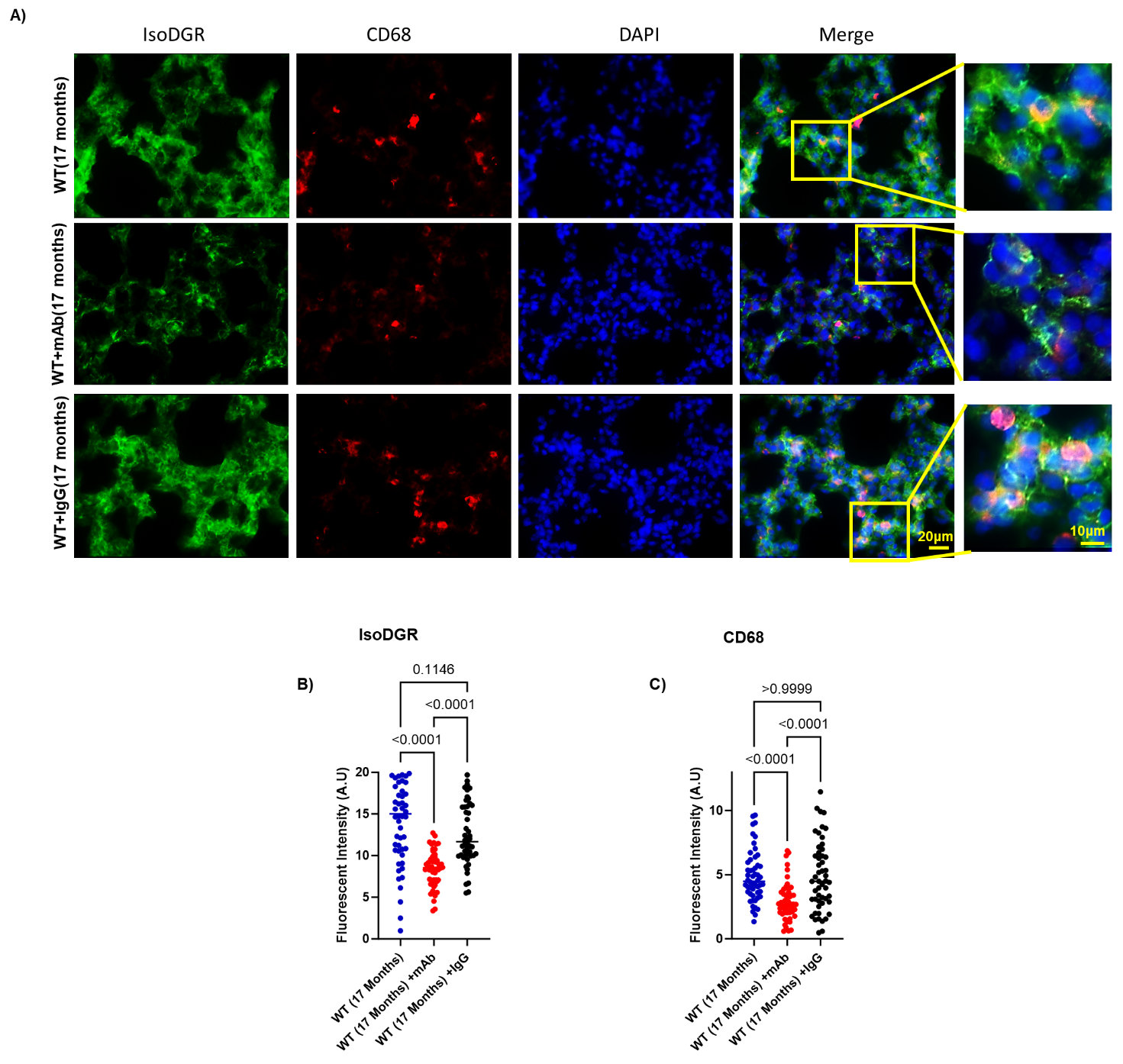


***Figure S9: Immune clearance of isoDGR-modified proteins in lung tissues of naturally-aged mice***

***(A)*** *Representative immunostaining images showing that isoDGR-proteins positive correlation with CD68^+^ macrophages in cryosectioned lung tissues of naturally-aged WT mice from isoDGR-mAb treated mice (compared with isotype IgG-injected controls)(n=5). Statistical significance was assessed using one-way ANOVA. Results are mean ± SEM.*


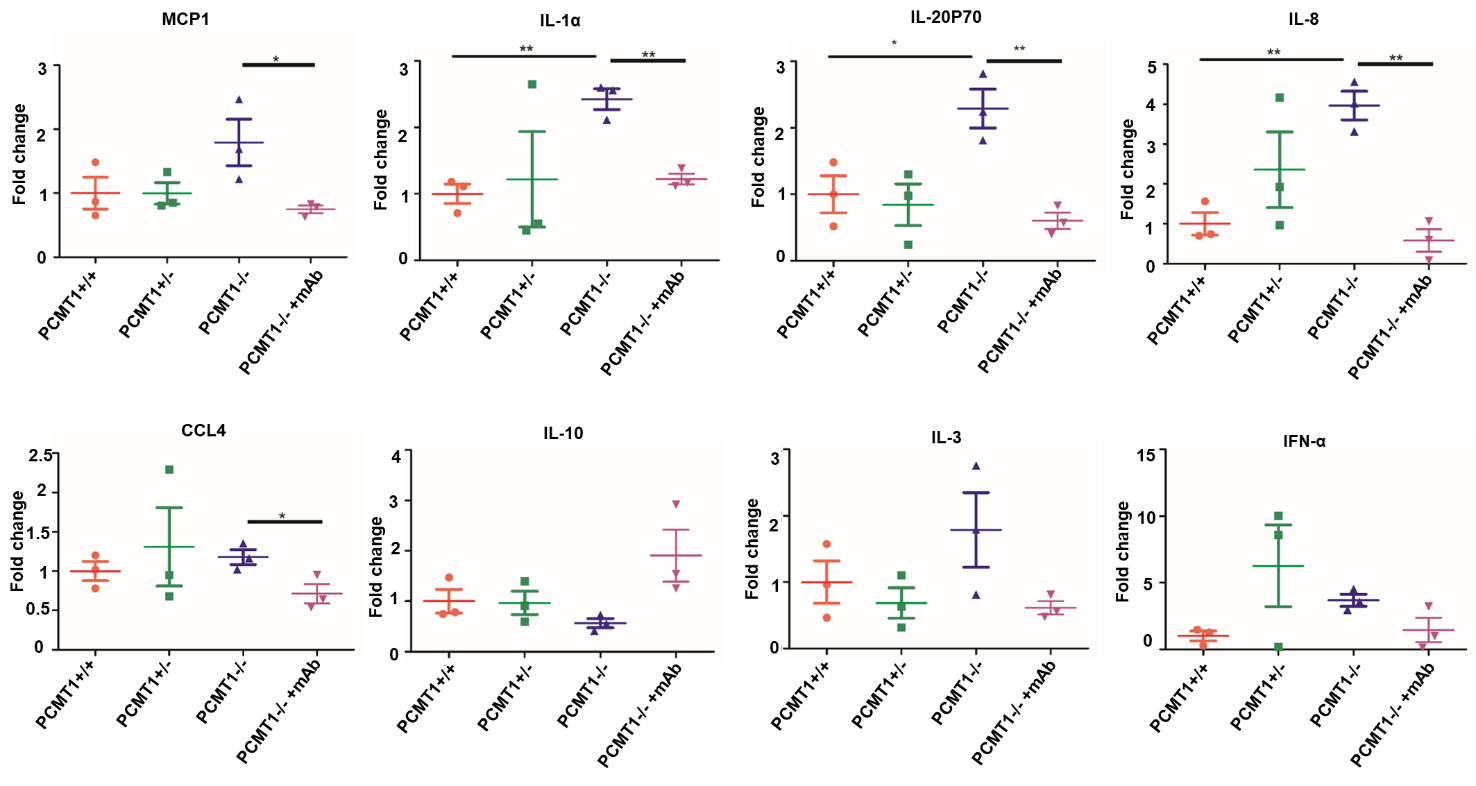


***Figure S10: Anti-isoDGR mAb reduces levels of pro-inflammatory cytokines in lung of Pcmt1^-/-^ mice*** *Graph represents quantitative PCR analysis of pro- and anti-inflammatory cytokine expression in lung tissue from Pcmt1^+/+^, Pcmt1^+/-^, Pcmt1^-/-^, and mAb-treated Pcmt1^-/-^ mice aged 5-6 weeks. Expression of GAPDH was used to normalize data (n=3). Statistical significance was assessed using one-way ANOVA. Results are mean values ± SEM (* p<0.05, ** p<0.01, *** p<0.001).*

**
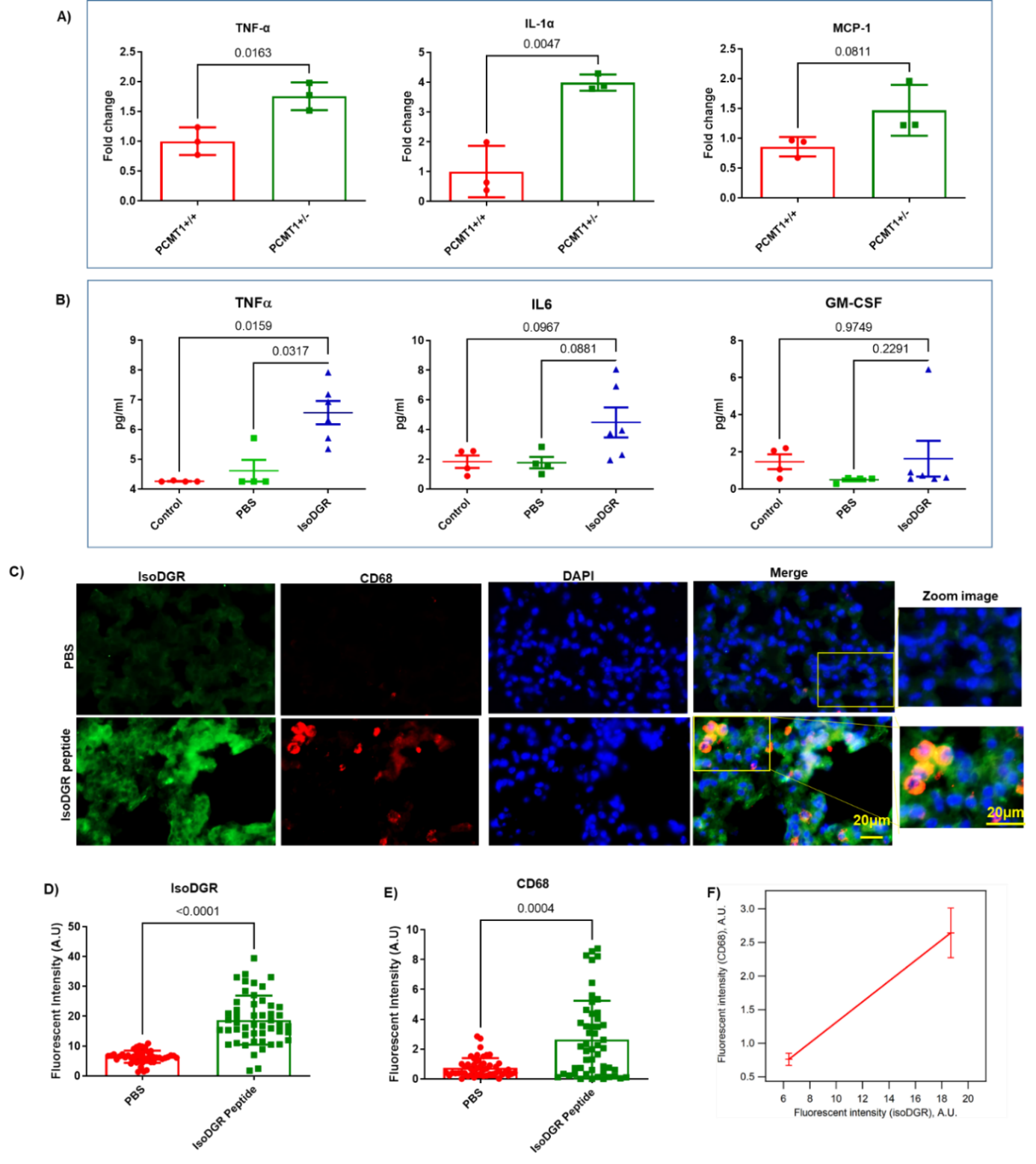
**

***Figure S11. Synthetic isoDGR-peptides induce lung inflammation that resembles natural aging***

***(A)*** *Graph shows quantitation of pro- and anti-inflammatory cytokine levels in lungs from 2 year-old Pcmt1^+/+^ and Pcmt1^+/-^ mice (n=3).* ***(B)*** *Concentrations of MCP1, IL-6, and GM-CSF in lung interstitial fluids from Pcmt1^+/+^ mice treated with synthetic isoDGR-peptide (n=6), or PBS-only vehicle control (n=4), or left untreated (n=4).* ***(C)*** *Representative immunostaining images showing that residual isoDGR peptide distribution co-localizes with CD68^+^ macrophages in cryosectioned lung tissue from treated mice (compared with PBS-injected Pcmt1^+/+^ controls)(n=4). Quantitative analysis of isoDGR-motif* ***(D)*** *and CD68 staining* ***(E)*** *in lung tissues.* ***(F)*** *CD68^+^ macrophage infiltration of lung tissues was positively correlated with isoDGR levels. Statistical significance was assessed using one-way ANOVA. Results shown are mean ± SEM.*


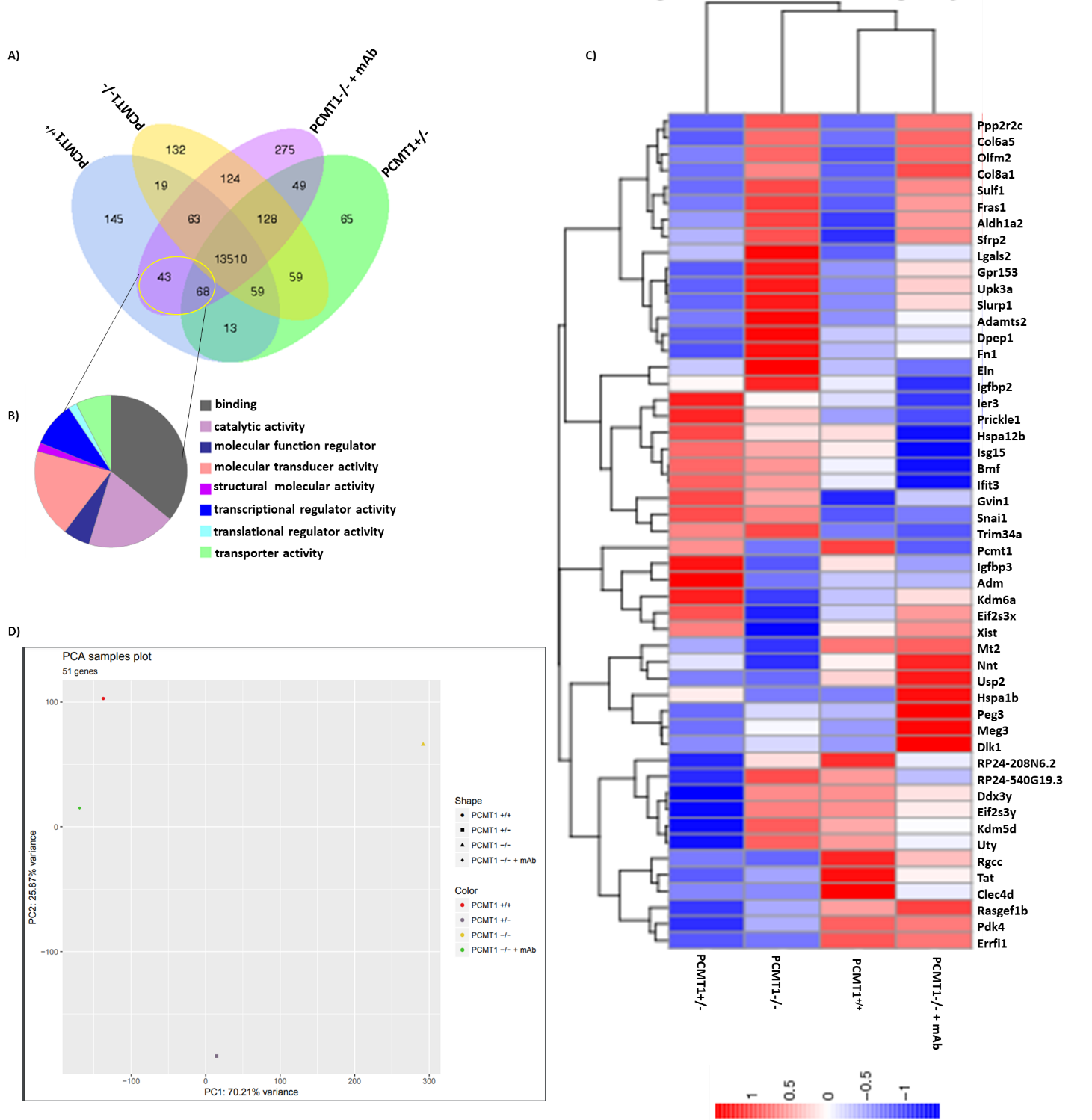


**
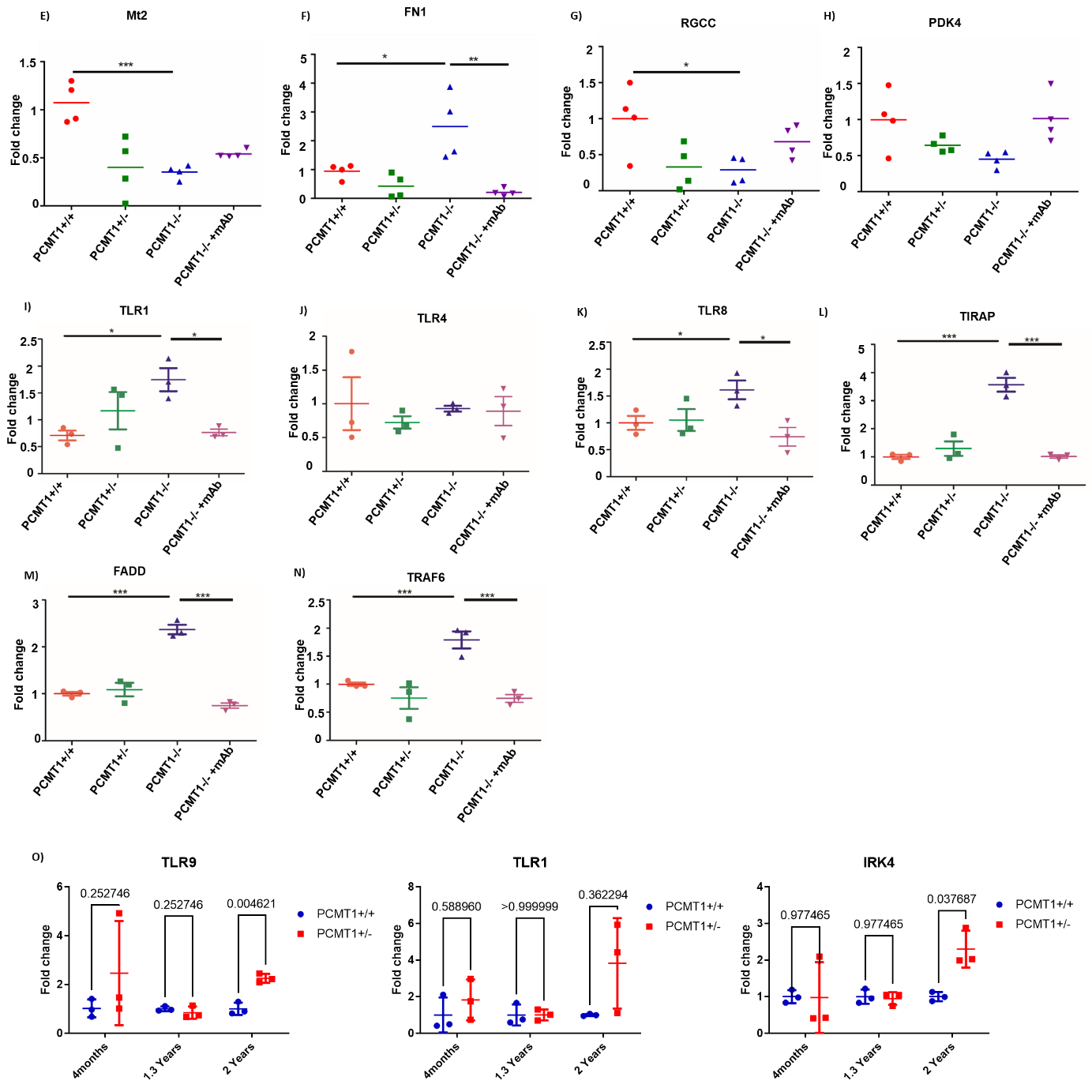
**

***Figure S12: Lung transcriptome of Pcmt1^+/+^, Pcmt1^+/-^ Pcmt1^-/-,^ and mAb-treated Pcmt1^-/-^ mice***

***(A)*** *Venn diagram indicating the number of differentially expressed genes across four samples (Pcmt1^+/+^, Pcmt1^+/-^, Pcmt1^-/-^ and mAb-treated Pcmt1^-/-^ mice) as well as overlap between each set of genes****. B)*** *Pie chart showing functional classification of 111 genes that were restored to normal expression levels in lungs from Pcmt1^-/-^ mice that received mAb treatment.* ***(C)*** *Hierarchical clustering of differential gene expression across all mouse lung samples in this study. Lung samples are represented by columns in the clustering heat-map and then sorted by genotype and treatment.* ***(D)*** *PCA plot for the 4 genotypes / treatment group. Each dot represents one group, color-coded according to genotype / treatment (Red:* *Pcmt1^+/+^; purple: Pcmt1^+/-^; yellow: Pcmt1^-/-^; Green: mAb-treated Pcmt1^-/-^). Graph shows quantitative PCR analysis of TLR signalling-associated genes TLR1* ***(E)****, TLR4* ***(F)****, TLR8* ***(G)****, TIRAP* ***(H),*** *FADD* ***(I)*** *and TRAF6* ***(J)*** *in lung from the four groups of mice at age 5-6 weeks.* ***(O)*** *TLR1, TLR9 and IRK4 relative expression in the lungs of Pcmt1^+/+^ and Pcmt^+/-^ mice at 4, 15, and 24 months old. Expression of GAPDH was used to normalize data. Statistical significance was assessed using one-way ANOVA. Results are mean ± SEM (* p<0.05, ** p<0.01, *** p<0.001).*


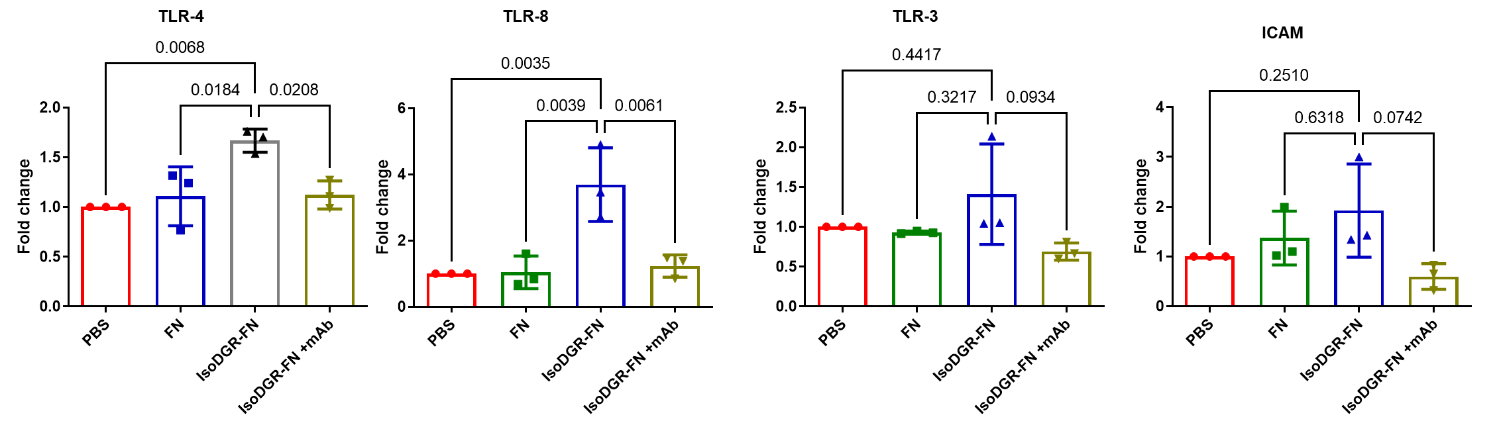


***Figure S13: IsoDGR-modified fibronectin activates TLR pathways*** *Graph showing quantitative PCR analysis of TLR-associated gene expression (TLR-4, TLR-8, TLR-3, and ICAM) in HUVEC cells cultured with FN or isoDGR-FN in the presence or absence of motif-specific mAb (or PBS-only control). Expression of GAPDH was used to normalize data (n=3). Statistical significance was assessed using one-way ANOVA. Results shown are mean values* *± SEM (* p<0.05, ** p<0.01).*


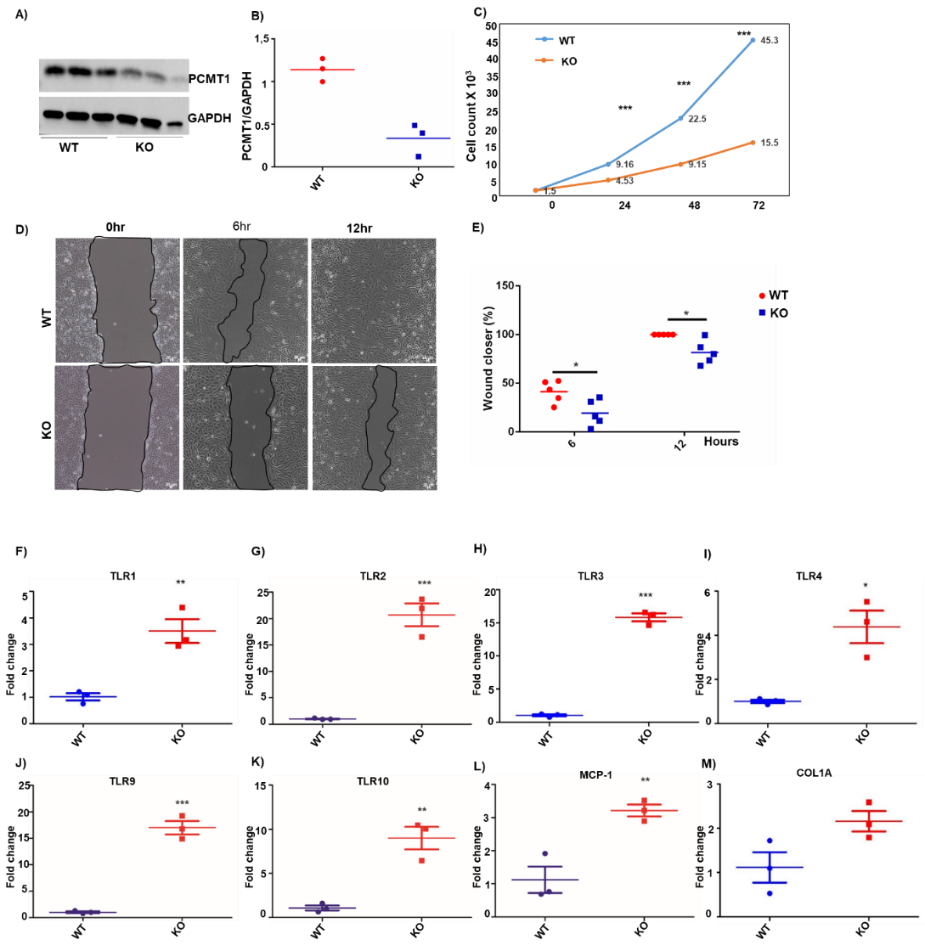


***Figure S14: Pcmt1 knock-down impairs HUVEC proliferation / migration and activates TLR pathways. (A, B)*** *Protein lysates from HUVECs and HUVECsPcmt1 ^KD^ were subjected to western blot analysis using antibodies against Pcmt1 and GAPDH loading control (n=3). Statistical significance was assessed using one-way ANOVA. Results shown are mean values ± SD (***p≤0.001).* ***C****) Graph showing proliferation rates of Pcmt1^+/+^ and Pcmt1-KO cells at 0, 24, 48 and 72h. (n=5)* ***D****) Representative images showing migration of HUVEC and HUVECPcmt1^KD^ cells (n=5)* ***E****) Graph in right panel shows quantitation of migration rate comparing HUVEC and HUVECPcmt1^KD^ (mean ± SD, n=3).* ***F-M****) Graphs show quantitative PCR analysis of TLR pathway-associated gene expression in HUVECs and HUVECsPcmt1 ^KD^. Data are normalised to GAPDH expression (n=3). Results are shown as mean values ± SEM (***p<0.001, **p<0.01, *p<0.05).*


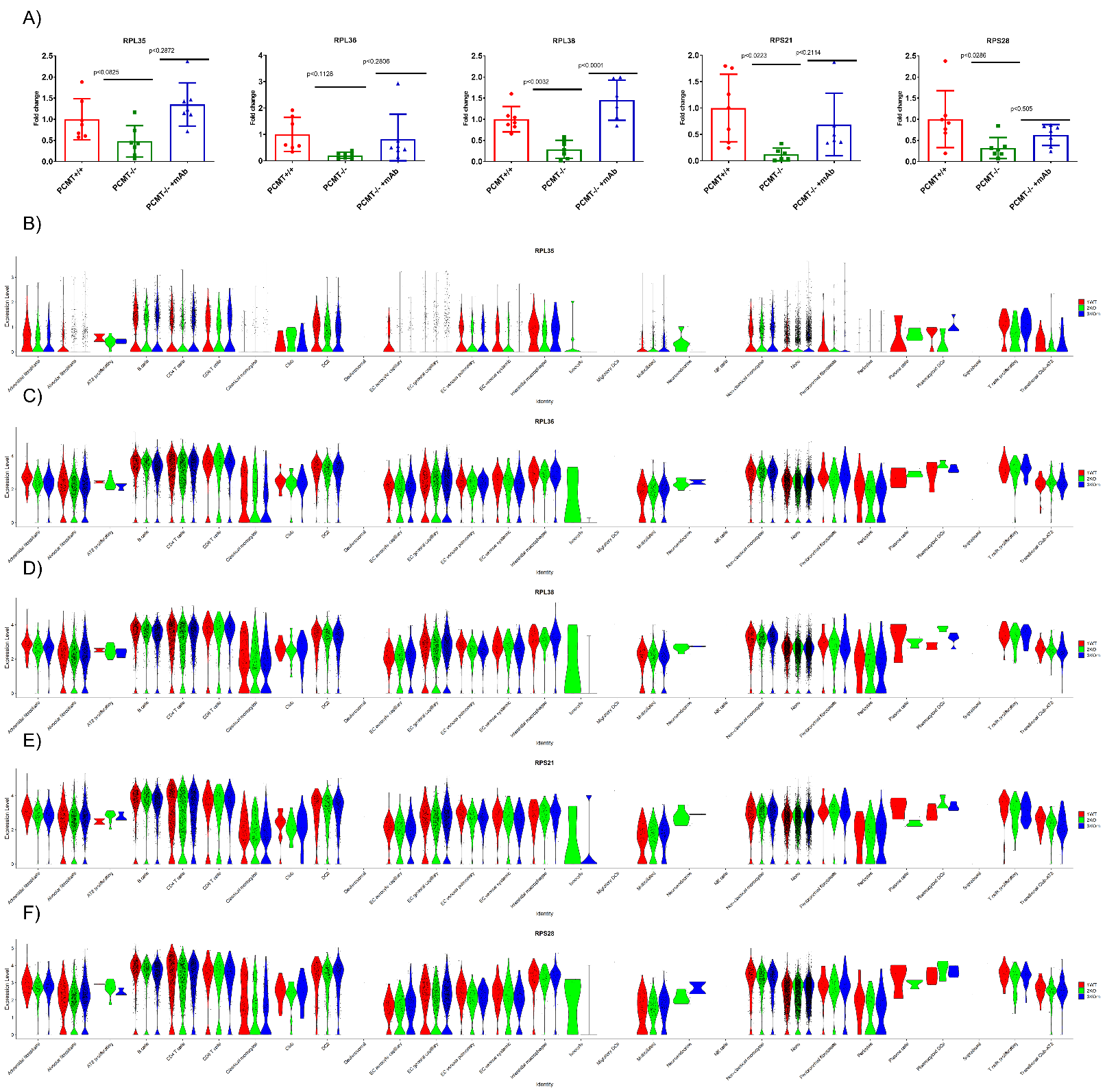


***Figure S15: IsoDGR mAb treatment increases expression of ribosomal synthetic machinery proteins in Pcmt^-/-^ mouse lung.*** *(A) Graph showing quantitative PCR analysis of ribosome-associated genes RPL35, RPL36, RPL38, RPS21, and RPS28 in lung tissues from Pcmt1^+/+^, Pcmt1^-/-^ and mAb-treated Pcmt1^-/^ mice at 5-6 weeks. Expression of GAPDH was used to normalize data. Statistical significance was assessed using one-way ANOVA. Results are mean ± SEM (n=6). Violin plots of (B) RPL35, (C) RPL36, (D) RPL38, (E) RPS21, and (F) RPS28 expression across various lung cell types, as determined by scRNA-seq analysis.*

*
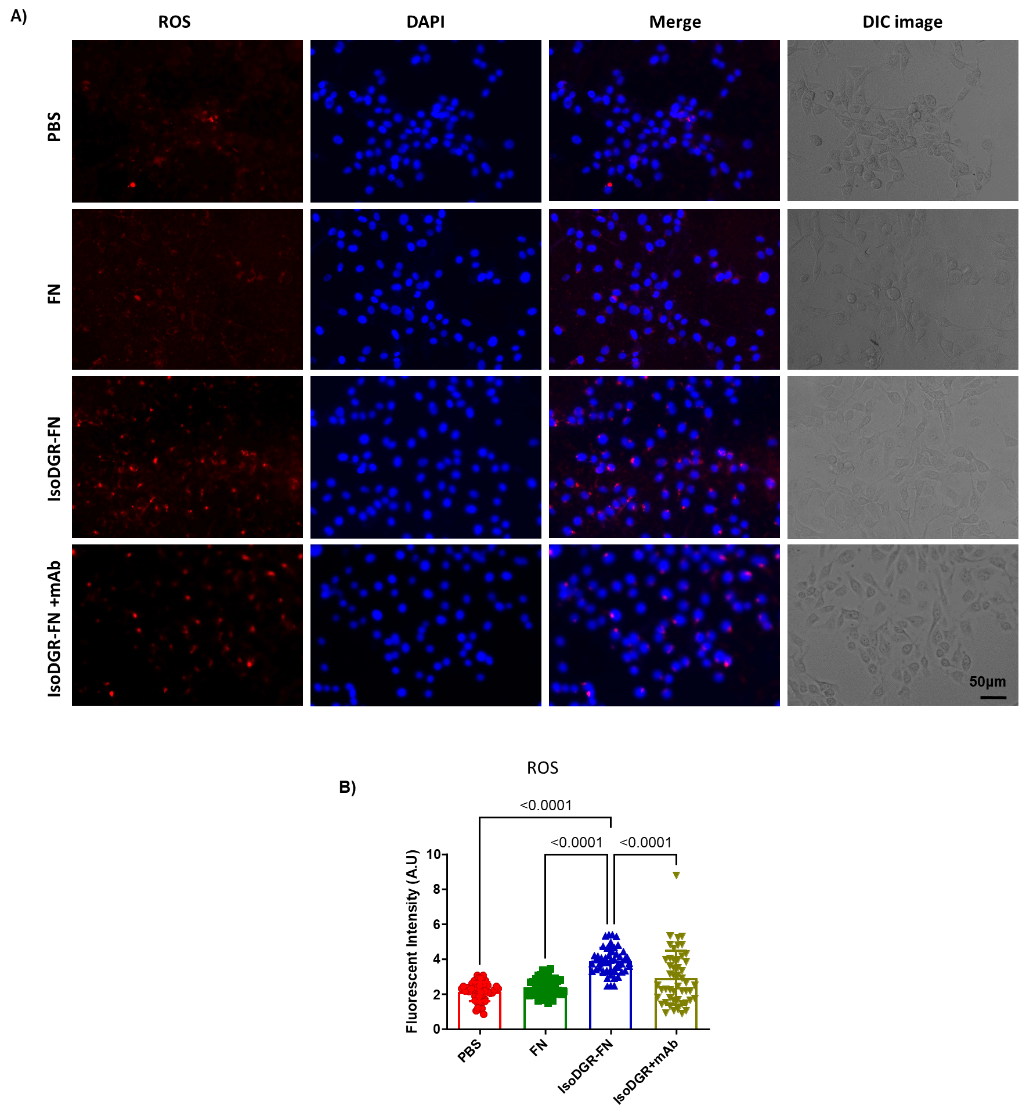
*

***Figure S16: IsoDGR-modified fibronectin increases ROS production in HULEC-5a cells***

***(A)*** *Representative immunostaining images showing ROS distribution in HULEC-5a cells cultured with PBS only, native FN, or isoDGR-FN, either in the presence or absence of anti-isoDGR mAb.* ***(B)*** *ROS fluorescence was quantified in Image J using 50 randomized regions from 5 images of 5 independent experiments (graphs show average values for the same region from 5 images). Statistical significance was assessed using one-way ANOVA. Results shown are mean values ± SEM.*

**
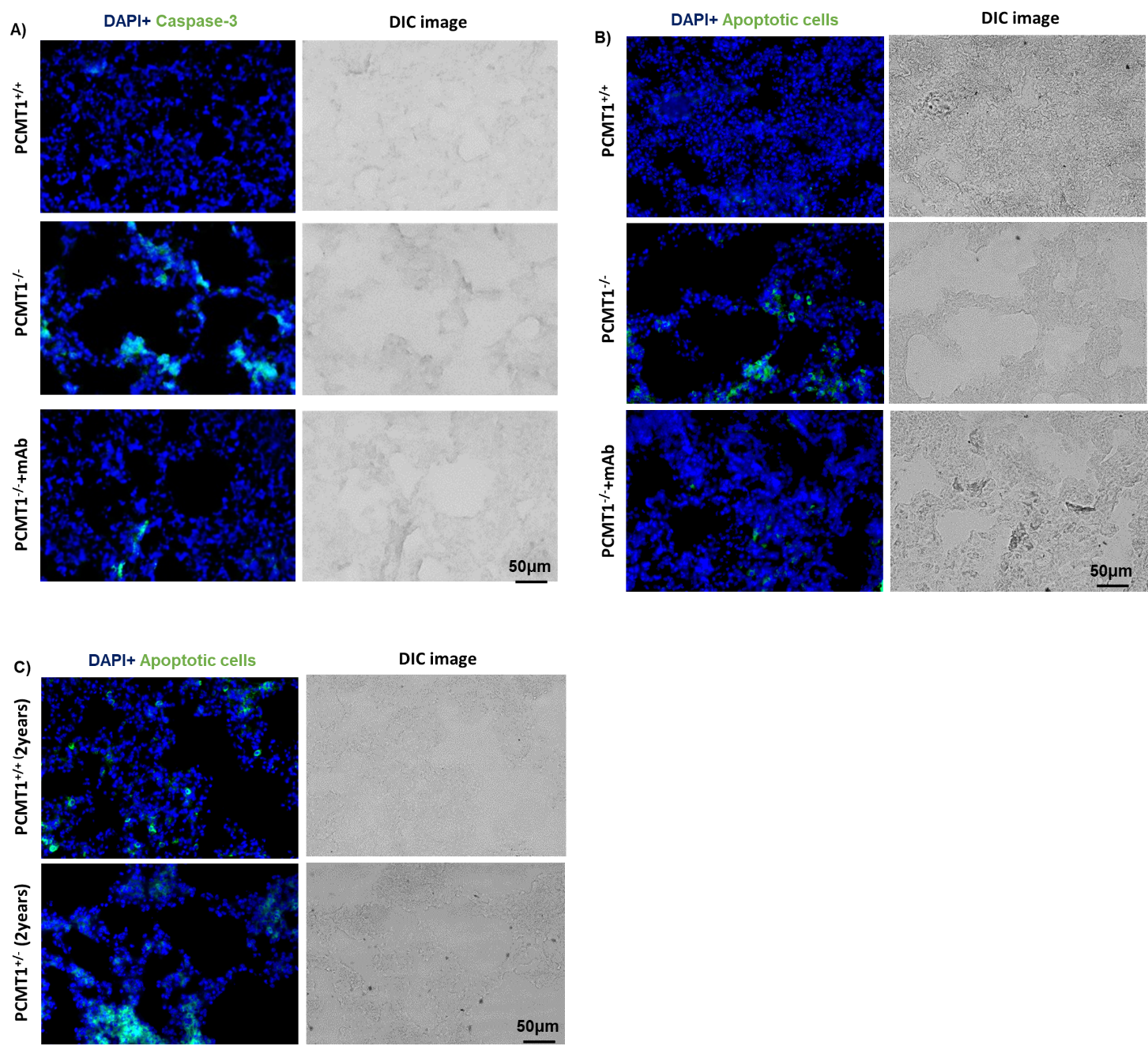
**

***Figure S17: IsoDGR-modified proteins induce apoptosis of lung parenchymal and EC cells***

*Representative images of Tunel assay identifying caspase-3 positive cells* ***(A)*** *and apoptotic cells (****B****) in lung tissue from* *5-6 weeks old Pcmt1^+/+^, Pcmt1^-/-^ and mAb-treated Pcmt1^-/-^ mice, or* ***(C)*** *in lung sections from 2-year-old Pcmt1^+/+^ and Pcmt1^+/-^ mice (n=3 per group).*


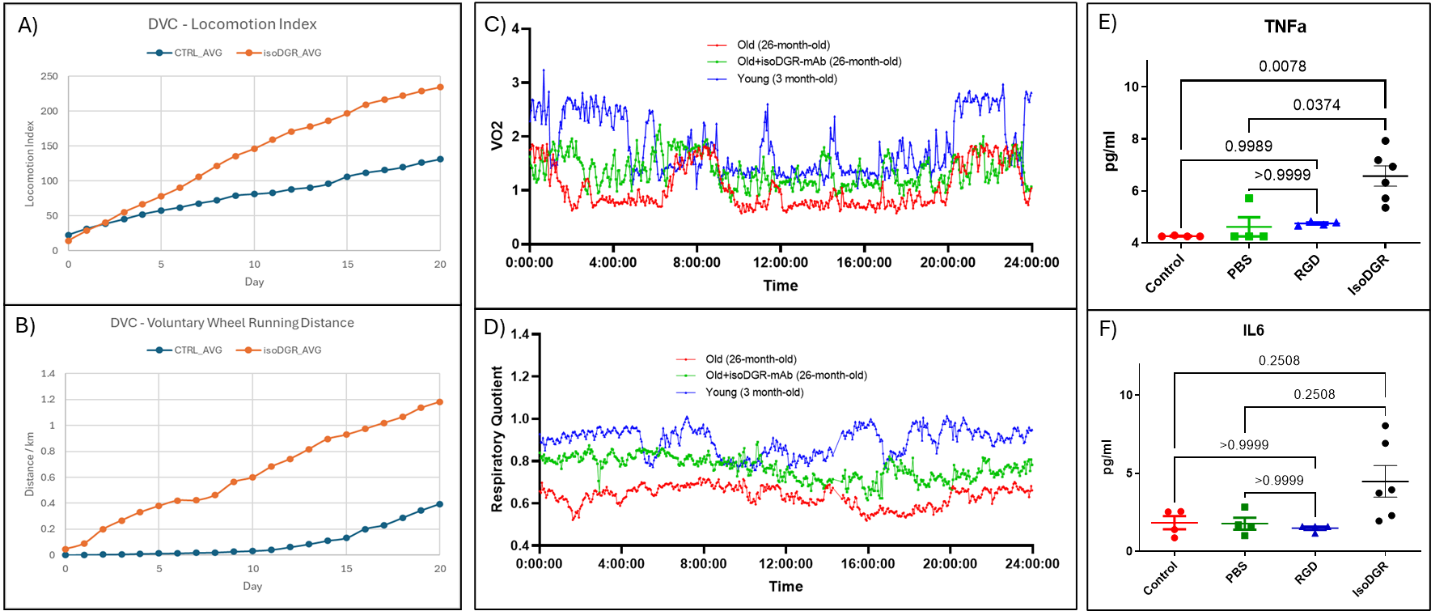


***Figure S18: Physical activity, exploration and respiratory quotient in old mice treated with anti-isoDGR and lung TNFa and IL6 profile in young mice.***

*A) Locomotion in Home Cage: Movement of mice in their home cages was recorded after isoDGR-mAb treatment (red) vs control). B) Voluntary Running Wheels (VRW): VRWs were introduced one-week post-treatment. mAb-treated 20-month-old mice (red) learned to use VRWs significantly faster and more frequently than age-matched control mice (blue). C) 24-hour VO2 Measurement: Oxygen consumption over 24 hours was measured in a metabolic cage for 3-month-old mice (blue), 26-month-old mice (red), and isoDGR-mAb-treated 26-month-old mice (green). D) Respiratory Quotient (RQ): metabolic cage measurement of RQ indicated a significant increase in the RQ for mAb-treated old mice. E) and F) TNFα and IL6 profiles in lung interstitial fluids of 6-week-old young mice in responses to intranasal RGD and isoDGR-peptides treatment. Statistical significance was assessed using one-way ANOVA(n=6). Results shown are mean values ± SEM.*

### Table S1 Primers used in genotyping mouse Pcmt1 gene.

| **Primer Name** | **Genotyping Primer** |
| --- | --- |
| oIMR108 | CGGCTGCATACGCTTGATC |
| oIMR1081 | CGACAAGACCGGCTTCCAT |
| oIMR1544 | CACGTGGGCTCCAGCATT |
| oIMR3580 | TCACCAGTCATTTCTGCCTTTG |

### Table S2 RTPCR Primers for mouse tissue

| **Gene name** | **Primer sequences** |
| --- | --- |
| **RPS28-F** | GCCGCTCTATCATCCGAAAT |
| **RPS28-R** | GCTGCAAGATTAACGCAACC |
| **RPL38-F** | GGTACCTTTACACCCTGGTTATC |
| **RPL38-R** | CAGAGGGCTGGTTCATTTCA |
| **RPL36-F** | AAACGTCAGTAAGCCGAGAC |
| **RPL36-R** | TGGACACTTTGAGCAACTCC |
| **RPL35A-F** | GCTGGGAACAGGACTTCTAAC |
| **RPL35A-R** | CCGTTTCATCTCGGGCATAA |
| **RPS21-F** | ACCACAGGCCGGTTTAATG |
| **RPS21-R** | GCCTTAGCCAATCGGAGAATAG |
| **TLR1-F** | GCTGGTGTTAGGAGATGCTTAT |
| **TLR1-R** | GACGGACACATCCAGAAGAAA |
| **TLR3-F** | GTGCATCGGATTCTTGGTTTC |
| **TLR3-R** | GACCCAGTCTCTGTCTTTATGG |
| **TLR4-F** | AGTATCGAGAGGCTCAGGTATAG |
| **TLR4-R** | TACAGGATGCAGGACAAGTAATC |
| **TLR8-F** | CCTCTCTAAGGCTAGGGTAACT |
| **TLR8-R** | TGCCCAGAAGACAGCATTT |
| **TIRAP-F** | CCATCATAGAGGTGGCTTTCC |
| **TIRAP-R** | TCGATGCCTTTATCTGCTACTG |
| **TRAF6-F** | GGTACCTGAGAGATGCCTAAAC |
| **TRAF6-R** | GACAGTCACCTCTCCACATTAG |
| **FADD-F** | CCTTTGGCTGGGTGTGATAATA |
| **FADD-R** | TCTCTATGTAGGCTAGGCTGTC |
| **MCP1-F** | TGATCCCAATGAGTAGGCTGGAG |
| **MCP1-R** | ATGTCTGGACCCATTCCTTCTTG |
| **IL-1a-F** | CAAACTGATGAAGCTCGTCA |
| **IL-1a-R** | TCTCCTTGAGCGCTCACGAA |
| **IL-8-F** | CGTGGCTCTCTTGGCAGCCTTC |
| **IL-8-R** | TCCACAACCCTCTGCACCCAGTT |
| **GAPDH-R** | AAGATGGTGATGGGCTTCCCG |
| **GAPDH-F** | TGGCAAAGTGGAGATTGTTGCC |
| **IL-3-F** | TGAAGGACCCTCTCTGAGGA |
| **IL-3-R** | CGCAGATCATTCGCAGAT |
| **CCL4-F** | CAAACCTAACCCCGAGCAACAC |
| **CCL4-R** | GGTCTCATAGTAATCCATCACAAAGC |
| **IL-10-F** | ATGCAGGACTTTAAGGGTTACTTGGGT |
| **IL-10-R** | ATTTCGGAGAGAGGTACAAACGAGGTTT |
| **IL-12p40-F** | CAGAAGCTAACCATCTCCTGGTTTG |
| **IL-12p40-R** | TCCGGAGTAATTTGGTGCTTCACAC |
| **TNF-α-F** | GCCTCTTCTCATTCCTGCTTG |
| **TNF-α-R** | CTGATGAGAGGGAGGCCATT |
| **TLR9-F** | TGGTTACCTGGCAAGACGC |
| **TLR9-R** | GGAAACTGGCACGCAAGAG |
| **IRAK-4-F** | CCATCGTGGCGGTGAAG |
| **IRAK-4-R** | GTGCTGACACGTTGCCATTACT |
| **mt-ND1-F** | GCTTTACGAGCCGTAGCCCA |
| **mtND1-R** | GGGTCAGGCTGGCAGAAGTAA |
| **mt-ND2-F** | CCTCCTGGCCATCGTACTCA |
| **mt-ND2-R** | GAATGGGGCGAGGCCTAGTT |
| **mt-ND3-F** | TAGTTGCATTCTGACTCCCCCA |
| **mt-ND3-R** | GAGAATGGTAGACGTGCAGAGC |
| **mt-ND4-F** | CGCCTACTCCTCAGTTAGCCA |
| **mt-ND4-R** | TGATGTGAGGCCATGTGCGA |
| **mt-ND4l-F** | AGCTCCATACCAATCCCCATCAC |
| **mt-ND4l-F** | AGCTCCATACCAATCCCCATCAC |
| **mt-ND5-F** | GGCCCTACACCAGTTTCAGC |
| **mt-ND5-R** | AGGGCTCCGAGGCAAAGTAT |
| **mt-ND6-F** | CTTGATGGTTTGGGAGATTGG |
| **mt-ND6-R** | ACCCGCAAACAAAGATCACC |
| **mt-Cytb-F** | TCCTTCATGTCGGACGAGGC |
| **mt-Cytb-R** | AATGCTGTGGCTATGACTGCG |
| **mt-CO1-F** | TCAACATGAAACCCCCAGCCA |
| **mt-CO1-R** | GCGGCTAGCACTGGTAGTGA |
| **mt-ATP6-F** | AGCTCACTTGCCCACTTCCT |
| **mt-ATP6-R** | AAGCCGGACTGCTAATGCCA |
| **mt-CO2-R** | TCCTAGGGAGGGGACTGCTC |
| **mt-CO2-F** | ACCTGGTGAACTACGACTGCT |

| **Gene Name** | **Primer sequence** |
| --- | --- |
| **TLR1-F** | CATGGCCAGGAGGACTTATTT |
| **TLR1-R** | TGCTTGCTCTGTCAGCTTAATA |
| **TLR2-F** | GAAGAGTGAGTGGTGCAAGTAT |
| **TLR2-R** | AATGGGCTCCAGAAGAATGAG |
| **TLR3-F** | CCCTGGTGGTCCCATTTATTT |
| **TLR3-R** | CTCAACTGGGATCTCGTCAAAG |
| **TLR4-F** | GATGAGGACTGGGTAAGGAATG |
| **TLR4-R** | GGCCACACCGGGAATAAA |
| **TLR8-F** | CACCAGAGACATAGGCATCAC |
| **TLR8-R** | TCGCATGGCTTACATGAGTATAG |
| **TLR9-F** | GCTAGACCTGTCCCACAATAAG |
| **TLR9-R** | AAAGGGCTGGCTGTTGTAG |
| **TLR10-F** | GCTAGACCTGTCCCACAATAAG |
| **TLR10-R** | AAAGGGCTGGCTGTTGTAG |
| **GAPDH-F** | GTGGTCTCCTCTGACTTCAACA |
| **GAPDH-R** | CTCTTCCTCTTGTGCTCTTGCT |
| **ICAM1-F** | CTCCAATGTGCCAGGCTTG |
| **ICAM1-R** | CAGTGGGAAAGTGCCATCCT |
| **MCP-1-F** | CAGATGCAATCAATGCCCCAG |
| **MCP-1-R** | ATAAAACAGGGTGTCTGGGGAAAGC |
| **COL1A1-F** | TCTGCGACAACGGCAAGGTG |
| **COL1A1-R** | GACGCCGGTGGTTTCTTGGT |

### Table S3 RT-PCR Primers for human cells

***Table S4: Linear Regression Results of Age-Dependent isoDGR Accumulation and Correlation of isoDGR with CD68 and CD11b in Human Lung Tissues (as shown in Figure 1)***

| **Independent Variable** | **Dependent Variable** | **Coefficient** | **P-value** | **R-squared** |
| --- | --- | --- | --- | --- |
| Age | isoDGR | 3.32 | 0.32 | 0.01 |
| isoDGR | CD68 | 0.16 | 1.02E-11 | 0.22 |
| isoDGR | CD11b | 0.09 | 1.58E-05 | 0.09 |

***Table S5: Linear Regression Results of Age-Dependent isoDGR Accumulation and Correlation of isoDGR with CD68 and CD11b in Fibrotic Human Lung Tissues (as shown in Figure S1)***

| **Independent Variable** | **Dependent Variable** | **Coefficient** | **P-value** | **R-squared** |
| --- | --- | --- | --- | --- |
| Age | isoDGR | 26.22 | 4.01E-03 | 0.15 |
| isoDGR | CD68 | 0.09 | 2.19E-02 | 0.10 |
| isoDGR | CD11b | 0.11 | 7.97E-03 | 0.13 |
